# Efficacy of a Web-Based Stress Management Intervention for Beginning Teachers on Reducing Stress and Mechanisms of Change: Randomized Controlled Trial

**DOI:** 10.2196/58475

**Published:** 2025-06-16

**Authors:** Hanna Heckendorf, Dirk Lehr

**Affiliations:** 1 Department of Health Psychology, School of Sustainability Leuphana University of Lüneburg Lüneburg Germany

**Keywords:** career starters, beginning teachers, internet-based intervention, indicated prevention, randomized controlled trial, RCT, stress, mechanisms of change, transactional stress theory

## Abstract

**Background:**

Teaching is often characterized as a stressful profession, with a substantial proportion of teachers experiencing chronic stress and burnout. Research indicates that teachers often experience stress upon entering the workforce, leading to detrimental effects on their health, occupational well-being, and work performance and adversely impacting student outcomes. While meta-analyses have demonstrated the efficacy of internet-based stress management interventions (iSMIs) for both experienced professionals and university students, there remains a gap in research on the efficacy of iSMIs tailored to teachers and career starters.

**Objective:**

We tailored an iSMI to beginning teachers and added a newly developed web-based classroom management skill training. This study examined the effectiveness of the tailored iSMI in reducing perceived stress and improving further secondary outcomes. In addition, this study was the first to explore problem-solving ability and emotion regulation skills as potential mechanisms of change within an iSMI grounded in transactional stress theory.

**Methods:**

Participants were 200 highly stressed beginning teachers (Perceived Stress Scale score of >21) undergoing German teacher induction randomized to either an intervention group (IG) with guidance or a waitlist control group. Outcomes were assessed online at baseline, 8 weeks after randomization (postintervention time point; T2), and at both the 3-month (T3) and extended 6-month follow-up. At T2, data were collected from 84% (84/100) of the participants in the IG and 90% (90/100) of the participants in the waitlist control group.

**Results:**

The IG reported significant, practically meaningful, and sustained improvements in both perceived stress (T2: *d*=0.52, 95% CI 0.24-0.80, and *P*<.001; T3: *d*=0.49, 95% CI 0.21-0.77, and *P*<.001) and most secondary outcomes in the intention-to-treat analysis using analyses of covariance. Postintervention (T2) effects on mental health indicators, including depression, work-related rumination, anxiety, and insomnia, were substantial (*d*≥0.60), whereas no significant effects were observed for absenteeism (*P*=.22) and presenteeism (*P*=.80). The investigated mediators, problem-solving ability (*d*=0.57) and emotion regulation skills (*d*=0.69), improved. Moreover, parallel mediation analysis revealed that the iSMI exerted its effect on perceived stress through improved problem-solving ability (indirect path a_2_b_2_=–0.77, 95% CI –1.50 to –0.04) and emotion regulation skills (indirect path a_1_b_1_=–0.97, 95% CI –1.73 to –0.22).

**Conclusions:**

This study contributes to the growing body of evidence on iSMIs for beginning teachers during a highly demanding transition phase and supports the integration of these interventions into teacher training programs. Findings from the mediation analysis suggest that both problem-focused and emotion-focused coping strategies should be equally considered in stress management interventions. This strengthens the program theory based on the transactional stress model. Finally, these findings encourage further research on iSMIs for other groups of career starters to support their transition into the workforce.

**Trial Registration:**

German Clinical Trials Register (DRKS) DRKS00013880; https://tinyurl.com/3mpsyvw5

## Introduction

### Stress in Teachers

#### Stress in Teachers and How It Begins

Being a teacher offers people the opportunity to make a worthwhile contribution to society; work with young people, including children; and shape the values of the next generation [1]. However, this positive outlook on the profession is often clouded. Teaching is repeatedly described as a stressful profession, with a significant proportion of teachers feeling chronically stressed [1-5]. A significant body of research has investigated the determinants of teachers’ stress and identified individual factors associated with teachers’ chronic stress and exhaustion that include inadequate active and passive coping and emotional instability [6,7]. Other contributory stressors in the working environment include high workload; demands concerning lesson preparation; and emotional demands due to, for example, student misbehavior [5,7-10].

When examining the onset of chronic stress, longitudinal studies suggest that chronic stress and emotional exhaustion begin during teachers’ entry into the workforce [11-17]. This transition from the academic to the working world has also been described as “practice shock” [18]. In Germany, this practice shock typically takes place during a highly structured trainee program that lasts 1.5 to 2 years—the *Referendariat*—which is when teachers gain their first comprehensive practical experiences. The *Referendariat* is an induction phase and a mandatory element of teacher education after university. During this period, beginning teachers gradually start to teach independently and undergo repeated examinations and evaluations of their teaching. At the same time, they attend courses on teaching [19]. After this, they enter the profession as fully licensed, in-service teachers.

Practice shock has been similarly described for career transitions and newcomer socialization across professions, both assumed to be associated with increased stress secondary to multiple uncertainties [20-25]. Longitudinal studies have revealed that employees’ first few years of employment are associated with increased instability in their well-being [26].

While some studies suggest that the initial increase in teachers’ emotional exhaustion tends to be followed by a decrease after the transition period [16,17], others have failed to detect any significant improvement following the transition period [12,14,15]. Instead, exhaustion levels tend to remain as high in established in-service teachers as in teachers at the end of their transition phase [14,15]. Assessing the trajectories of change in a 3-year longitudinal study of beginning teachers, Hultell et al [27] failed to delineate any universal development of chronic stress. While, for some, burnout symptoms appeared to decrease after 1 to 2 years, for many others, they either remained stable at a moderate or high level or continued to increase. Similar patterns of heterogeneous burnout development have also been observed among career starters in nonteaching professions [28,29]. This highlights how the transition into working life can be challenging for many career starters and that this can be associated with further increases in stress levels that, for many individuals, will not diminish over many years.

#### Adverse Consequences of Stress for Teachers’ Health

Chronic work-related stress across professions is a known risk factor for adverse mental and physical health. With regard to mental health, several meta-analyses of prospective studies have revealed that an increased risk of chronic work-related stress leads to mental health problems such as anxiety, depression, stress-related mental disorders, and sleeping problems [30-33]. Regarding physical health, meta-analyses have also found work-related stressors to be associated with increased risks of coronary heart disease [34,35], metabolic syndrome [36], and unhealthy lifestyles [37].

Although the aforementioned results generally stem from studies examining the general working population, there is evidence from individual, mostly cross-sectional studies examining teachers suggesting that such relationships also apply to this population. For example, studies on teachers show high job strain to be associated with decreased recovery following work due to poorer sleep quality and increased work-related rumination [5,38,39]; with symptoms of depression, anxiety, and burnout [40-44]; with psychosomatic symptoms [45]; and with major depressive disorder [46]. Regarding physical health, individual studies on teachers show an association between high (vs low) job strain and factors linked to increased cardiovascular risk, including increased physiological stress responsivity and sustained high blood pressure [47-49].

#### Adverse Consequences of Stress on Teachers’ Work Productivity

In addition to consequences affecting teachers’ individual health and well-being, chronic stress is also presumed to influence teachers’ work performance and work behavior [50-52], with empirical evidence linking chronic stress in teachers to poorer work engagement, lower organizational commitment [9], and diminished job satisfaction [53]. Chronically stressed teachers also show higher rates of absenteeism and presenteeism [54], as well as greater intention to quit and teacher attrition [55]; moreover, this can be observed both in beginning teachers [56] and experienced teachers who retire early [57].

#### The Impact of Teachers’ Stress on Education

The societal consequences of stress go beyond the health care system, exerting an impact on the education system as well. With regard to teachers’ performance in the classroom, in a recently published study by Klusmann et al [58], teachers’ reduced instructional quality mediated the negative effect of their emotional exhaustion on a range of student outcomes. There is also a growing body of evidence suggesting that poorer teacher mental health is associated with lower student perceptions of the learning climate and teacher support, less school satisfaction, and reduced motivation and self-concept [58-60]. Poorer teacher mental health also appears linked to lower student achievement on standardized tests [58,59,61] and deterioration in students’ mental health and well-being [62,63].

To conclude, the aforementioned findings underscore the importance of preventing chronic work-related stress in teachers both for teachers’ health and well-being and for students’ academic and socioemotional development and well-being. Past research also suggests that chronic work stress begins at the very beginning of many teachers’ career and often does not decrease over time. Thus, developing stress management interventions (SMIs) for beginning teachers as a means to reduce perceived stress and prevent stress-related symptoms and disorders right from the beginning is crucial.

### Occupational Internet-Based Interventions

#### Previous Evidence on Occupational Internet-Based Interventions

Over the last 2 decades, research on the efficacy of internet-based SMIs (iSMIs) has grown and includes research on delivering interventions using the internet to enhance workers’ mental health [64]. Internet interventions are “treatments that have been operationalized and transformed for delivery via the Internet. Usually, they are highly structured, self- or semi-self-guided, based on effective face-to-face interventions, personalized to the user, interactive, enhanced by graphics, animations, audio, and possibly video and tailored to provide follow-up and feedback” [65]. Compared to traditional SMIs, iSMIs are usually conducted individually instead of in groups; they are also readily accessible and can be conducted in a self-paced manner without disclosing personal information to colleagues. Thus, iSMIs may overcome several barriers to traditional SMIs [64].

However, to date, no randomized controlled study has assessed the efficacy of an iSMI in beginning teachers. Moreover, reviewing the state of research on internet-based interventions for teachers in general, Lehr et al [66] identified no intervention primarily addressing stress that was evaluated solely in teachers. Although evidence assessing the efficacy of iSMIs in teachers remains scarce, meta-analyses have demonstrated the efficacy of iSMIs in reducing perceived stress and other mental health symptoms, such as depressive and anxiety symptoms, in university students [67,68], as well as in employees averaging 2 decades of job experience [69-71]. However, there remains a lack of studies investigating the efficacy of iSMIs during the challenging period of career start in both teaching and other professions.

#### Tailoring Occupational Internet-Based Interventions to Beginning Teachers

Although iSMIs for employees have been found to be effective in reducing stress in the general working population, meta-analyses have identified considerable heterogeneity between interventions [69-71] that might be explained by a suboptimal fit between the intervention and the stressors specific to the job of interest. Similarly, following their meta-analysis, Iancu et al [72] concluded that considering stressors specific to the teaching environment could improve the efficacy of interventions targeting teachers’ mental health. Tailoring internet-based interventions to individuals’ characteristics and needs has also been suggested as a possible way to increase the person-intervention fit and reduce the typically high attrition rates observed for such interventions [64,73]. Discussing clinical interventions, Schaeuffele et al [74] distinguished between unified and individualized intervention approaches. While unified approaches refer to untailored interventions that, as a whole, are applied to a range of problems as a “broadband” intervention, individualized approaches are tailored to individuals’ specific problems and needs. For iSMIs, a unified approach would refer to modules fostering more general stress-coping skills, whereas an individualized approach could be implemented by offering tailored optional modules for more individual problems. Tailored optional modules for individual problems might focus, for example, on stressors among particular employee groups or occupations or during specific career phases. Thereby—in addition to fostering more general stress-coping skills—these modules could help users apply general stress-coping skills to alleviate specific occupation- or phase-specific stressors.

A major occupation-specific stressor for teachers in general, and beginning teachers in particular, is students’ disruptive behavior, and coping with this stressor requires specific professional problem-focused coping skills [10,56,75]. Such classroom management (CRM)–specific coping skills are lower in beginning than in more experienced teachers [53,76,77] and have been shown to both predict reduced classroom disturbances and buffer the effects of classroom disturbances on emotional exhaustion [19,78,79]. The stress-reducing effects of promoting professional skills in career starters have been reported by Dicke et al [80], who investigated a CRM intervention in a nonrandomized trial involving beginning teachers. However, to the best of our knowledge, no trial has yet tailored an iSMI by adding occupation-specific individualized modules or, more specifically, modules to increase CRM-specific problem-solving skills.

#### Intervention Theory and Mechanisms of Change

To date, research on iSMIs has focused on efficacy in reducing stress. At the same time, the theory underlying the intervention’s design has remained largely underinvestigated, a pattern often observed in complex interventions that hinders theoretical progress [81]. Although current research suggests that theory-based interventions are not necessarily more effective than non–theory-based interventions, “theory provides a coherent and explicit framework for designing, evaluating, and optimizing interventions...and a means for accumulating evidence over time” [82]. Accordingly, successive studies that refer to a common theoretical framework and use the same language may lead to the systematic accumulation of knowledge so that theory-based interventions might be more easily improved and refined in the future and, ultimately, become more effective than non–theory-based interventions.

Knowing whether an intervention’s theorized program elements actually exert their effect on the outcome as a mechanism of change could aid in the development, refinement, and tailoring of successful interventions and theory [82,83]. Understanding these mechanisms might also be helpful to identify moderators of intervention efficacy and potentially allow for a better match between interventions and individuals [83].

In the transactional stress theory by Lazarus and Folkman [84], stress is seen as a result of an individual’s appraisal of an event and way of coping. When confronted with a potentially stressful event, an individual evaluates its relevance (primary appraisal) and the resources they have available to manage that event (secondary appraisal). For an event to be considered stressful, an individual must perceive it as dangerous and the resources available as insufficient to deal with it. To overcome the stress that has arisen, Lazarus and Folkman [84] distinguish between problem-focused and emotion-focused coping. This differentiation provides a useful framework with which to develop iSMIs.

Problem-focused coping “is directed at managing or altering the problem causing the distress” [84] and has been shown in meta-analyses to be associated with less psychopathology, including anxiety and depressive symptoms [85]. Adopting the definition of Lazarus and Folkman [84], CRM represents a specific problem-focused coping strategy as it aims to manage or change distress caused by challenging classes and students. Meta-analyses have also documented the efficacy of training on problem-focused coping in enhancing mental health [86].

Meanwhile, emotion-focused coping “is directed at regulating [the] emotional response to the problem” [84]. With the third-wave of cognitive behavioral therapy, strategies such as mindfulness and acceptance [87,88] have been argued to be adaptive emotion-focused coping strategies and linked to improved mental health [85,89-92]. Recent research suggests that the ability to flexibly use both problem-focused and emotion-focused coping strategies is important [93,94]. While Lazarus and Folkman [84] assumed that emotion-focused skills are helpful for addressing relatively unsolvable problems, they assumed that problem-focused coping is stress reducing with relatively controllable problems. Thus, in a complex world, the dynamic use of both strategies seems to be adaptive for successful coping.

Following this line of reasoning, Heber et al [95] developed an iSMI for the general working population that incorporates 3 sessions targeting problem-focused coping and 3 sessions addressing emotion-focused coping. Although the iSMI’s effectiveness has been demonstrated in several randomized controlled trials (RCTs) [95-100], this cannot be interpreted as proof of the theoretical assumptions made as knowing that an intervention works does not necessarily explain why it works.

To the best of our knowledge, no study has investigated whether teaching problem-focused and emotion-focused coping strategies within an iSMI alters particular coping orientations or whether these 2 approaches together mediate an iSMI’s effect on stress.

In other words, we do not know whether teaching both types of coping strategies leads to reduced perceived stress by changing both types of coping orientations.

### Aims of This Study

This study had 2 primary aims. The first was to assess the efficacy of an iSMI tailored to beginning teachers struggling with their transition into working life. Following the CONSORT (Consolidated Standards of Reporting Trials) [101,102] guidelines, this primary outcome was distinguished from further exploratory secondary outcomes. We hypothesized that the iSMI would decrease perceived stress as the primary outcome. Furthermore, it was hypothesized that the iSMI would also improve secondary outcomes that included mental and work-related health outcomes (eg, depression, anxiety, and work-related rumination), coping orientations, and self-efficacy.

The second aim was to assess whether an iSMI grounded in transactional stress theory would help reduce perceived stress by fostering problem-solving ability and emotion regulation skills. We hypothesized that problem-solving ability and emotion regulation skills are mediators of change influencing the intervention’s effect on perceived stress.

## Methods

### Study Design

A 2-arm RCT was conducted comparing the efficacy of an iSMI tailored to beginning teachers (intervention group [IG]) against a waitlist control group (WLG). The intervention was investigated as a measure of indicated prevention. Participants in the WLG obtained access to the intervention after the 3-month follow-up. During the study phase, participants in both groups were allowed to seek any treatment outside the study. Thus, participants had unrestricted access to all health care services typically offered in the German health care system (care as usual). However, before the study phase, both (1) being on a waiting list to receive and (2) currently receiving psychotherapy were exclusion criteria, but during the study, participants were permitted to make use of it.

In accordance with the CONSORT guidelines (Multimedia Appendix 1) for parallel-group randomized trials [101], we prespecified a primary outcome and secondary outcomes.

On the basis of the effect sizes found in a meta-analysis for internet-based interventions for perceived stress [69], as well as the effect size found for a previous version of the training program that only included the stress management module and was conducted in the general working population [95], a between-group effect size of *d=*0.4 at 8 weeks after randomization (T2) was anticipated. An effect of that size reflects a difference of 2.45 points on the Perceived Stress Scale (PSS-10) assuming an SD of 6.12 (pooled SD at T2 in the study by Heber et al [95]). Any difference (Δ) exceeding 2 points on the PSS-10 is considered clinically meaningful [103]. To detect an effect of that size, an a priori power analysis for a 2-tailed test with a power (1 – β) of 0.80 and significance level of .05 indicated a required sample size of 200 individuals.

### Participants and Procedures

All participants were beginning teachers in Germany taking part in the *Referendariat* (see the Introduction section for a more detailed description). Individuals were recruited both directly (through social media groups for beginning teachers on Facebook and Instagram) and indirectly by contacting the beginning teachers’ mandatory study seminars and asking the organizers to share information about the study. Through these channels, a flyer was distributed to beginning teachers providing them with information regarding stress during the *Referendariat*, the SMI under study, and a link to the study’s landing page to express their interest in participating.

Recruitment took place between August 2018 and May 2019. Interested individuals were sent a link to further information about the conditions of participation as well as the processing and handling of personal data. After the beginning teachers provided their informed consent online, the following inclusion criteria were assessed in a screening questionnaire: (1) current participation in the *Referendariat*; (2) elevated perceived stress, as indicated by a score of >21 on the PSS-10 to reflect an indicated preventative setting [104]; (3) not on a waiting list to receive or currently receiving psychotherapy; (4) no changes in the dosage of any psychopharmacological treatment over the preceding 30 days; (5) no reported acute suicidal tendencies (score on the suicidality item from the Beck Depression Inventory–II of <2 [105]); and (6) no past diagnosis of psychotic or dissociative symptoms.

Eligible individuals were then sent a link to the baseline questionnaire (T1). After they completed the baseline questionnaire, participants were randomly assigned to 1 of the 2 study arms. Randomization was conducted anonymously without any personal contact between study personnel and participants using a computer-generated randomization list with a ratio of 1:1 and block size of 2. The list was generated by a member of our department who was not otherwise involved in the study.

Blinding to group allocation was infeasible as participants were aware of whether they were randomized to the IG or the WLG. Participants in the IG received immediate access to the training program along with a message from an e-coach providing information about available guidance and how it is delivered. Individuals in the WLG were granted access to the program after they completed the 3-month follow-up.

### Study Conditions

#### Waitlist Control Arm

Participants in the WLG were offered access to the training program after the 3-month follow-up. During the study period, participants in both groups had unrestricted access to usual care services outside the study.

#### Intervention Arm

Participants in the IG received immediate access to the internet-based intervention, which was called “Starting calm through the induction phase.” This iSMI consisted of general information, interactive and writing exercises, quizzes, pictures, videos, and audio files and was specifically tailored to beginning teachers within the German induction phase. Throughout the intervention, participants were “accompanied” by 3 fictitious characters to facilitate social learning. These characters were also beginning teachers within the induction phase who shared information about their difficulties, how they completed the exercises, and what helped them. Every participant received their own password-protected learning environment to which they logged in and where their entries were saved. Homework was assigned between the sessions so that users could integrate what they had learned into their daily life. The training program was an iSMI that combined mandatory unified and optional individualized modules [74] to offer users a flexible intervention so that they could choose to hone specific skills to meet their personal needs.

The unified modules of the iSMI included 7 weekly sessions, each averaging 45 to 60 minutes in duration. The iSMI was developed based on the transactional model of stress by Lazarus and Folkman [84] with the aim of fostering 2 main coping strategies: problem-focused coping and emotion-focused coping.

In session 1, participants received psychoeducation on stress. In sessions 2 and 3, the importance of problem-solving was emphasized. Participants were introduced to a 6-step process to systematically solve problems. An advantage of this method is that participants were able to address almost all kinds of stressors they were confronted with. The aims were to reduce dispositional avoidance of stressors and participants’ negative problem orientation.

In sessions 4 to 6, participants learned different emotion-focused coping skills to help them foster both the understanding and acceptance of difficult emotions, as well as emotional self-support when they felt stressed.

In session 7, participants could generate a plan for their future use of the coping strategies they had learned.

Moreover, in each session, participants were given information on the importance of engaging in daily positive activities and were asked to plan them for the upcoming week.

The individualized modules contained optional information and solution approaches for both occupation-unspecific (eg, psychological detachment from work, sleep hygiene, worrying, and breaks) and teacher-specific stress-related problems. While the individualized modules on occupation-unspecific problems were also part of previous RCTs [95], for this RCT, the teacher-specific modules targeting different aspects of CRM were newly developed (eg, classroom design, establishing rules and routines, disruption-preventive CRM, and how to manage disruptive behavior) as CRM problems have been documented to be a key stressor for many—but not all—beginning teachers [53,76,77]. A more detailed description of the iSMI can be found in both Table 1 and a previous publication by Heber et al [95].

**Table 1:** Session content of the stress management intervention (see also the study by Heber et al [95] for a more detailed description of the stress management intervention) complemented by the optional individualized modules^a^.

Session	Objectives	Exercises
Psychoeducation	Introduction and provision of basic information on stress	Quiz on stress Stress analysis, stress diary, and identification of personal stressors Definition of goals and motivation for the training Planning of positive activities
Problem-focused coping I	Enhancing problem-focused coping skills	Stress analysis Differentiation between controllable and uncontrollable stressors Working on a 6-step problem-solving plan
Problem-focused coping II	Enhancing problem-focused coping skills	Taking stock of the 6-step problem-solving plan from the previous session and either continue working on it or work on a new 6-step problem-solving plan Identifying obstacles to training and ways to overcome them
Emotion-focused coping I	Learning muscle and breathing relaxation	Taking stock of the 6-step problem-solving plan Learning muscle and breathing relaxation techniques using an audio file
Emotion-focused coping II	Fostering understanding and acceptance of one’s unpleasant emotions	Finding the positive function behind unpleasant emotions; working on a 5-step plan to accept and tolerate unpleasant emotions and practicing it using an audio file
Emotion-focused coping III	Fostering emotional self-support	Strengthening positive self-evaluations, self-care, and self-compassion
Plan for the future	Reflecting on helpful strategies and planning them for the future	Choosing valuable strategies for the future

^a^Optional individualized modules: (1) occupation-unspecific problems (time management, rumination and worrying, psychological detachment from work, sleep hygiene, nutrition and exercise, organization of breaks during work, and social support) and (2) beginning teacher–specific problems regarding classroom management (teacher-student relationship, classroom design, establishing rules, establishing routines, disruption-preventive classroom management, and managing problem behavior).

Throughout the training, participants received personalized, written feedback on every completed session from an e-coach (undergraduate psychology students previously trained and continuously supervised by the first author). e-Coaches also sent users up to 3 reminders for them to complete the following session when it was not completed within 10 days of finishing the previous session. Feedback provision was manualized. Feedback was provided within 48 hours of a session being completed. Each feedback message consisted of roughly 500 words sent via email. Each of these messages was worded in a validating, motivating, and supportive way. The e-coaches were instructed to spend an average of 30 minutes creating 1 feedback message.

### Measures

#### Overview

Data collection took place at the time of screening (T0), at baseline (T1), immediately after the intervention (8 weeks after randomization; T2), and at the 3-month follow-up in both groups. For participants in the IG, outcomes were also assessed via an additional 6-month follow-up. Demographic variables were collected at T0. Outcomes measuring participants’ satisfaction with the training program and use of care as usual were assessed at T2.

Internal consistencies for this study are reported for T1 unless otherwise stated. Table S1 in Multimedia Appendix 1 provides the internal consistencies of the variables at all assessment points. All instruments were self-report measures assessed online and written in German.

#### Primary Outcome Measure

The PSS-10 [104] was used to measure general perceived stress. This scale consists of 10 items (eg, “In the last month, how often have you felt nervous and “’stressed’?”). Items are rated from 0 (never) to 4 (very often). The scale’s total score ranges from 0 to 40, with higher scores indicating greater perceived stress. Any score of >21 indicates an above-average level of stress [95]. The PSS-10 has good psychometric properties, as demonstrated by a Cronbach α ranging from 0.74 to 0.91 [106]. The Cronbach α was 0.71 at T1 in this study.

#### Secondary Outcome Measures

##### General Mental Health Outcomes

###### Symptoms of Depression

Symptoms of depression were measured using the short version of the Center for Epidemiological Studies Depression Scale, which consists of 15 items (eg, “During the last week I was depressed”), each rated from 0 (rarely or not at all) to 3 (most of the time or all the time). Internal consistency has been reported to be high, with the Cronbach α ranging between 0.87 and 0.92; it was 0.89 in this sample. Any total score of ≥18 indicates a clinically significant level of depression [107-109].

###### Symptoms of Anxiety

Generalized anxiety symptoms were measured using the 7-item version of the Generalized Anxiety Disorder Scale [110], with each item (eg, “Over the last 2 weeks, how often have you been bothered by the following problems...Feeling nervous, anxious or on edge”) rated from 0 (not at all) to 3 (nearly every day). Internal consistency is reported to be high (Cronbach α=0.92; 0.80 in this sample). A score of ≥10 indicates moderate to severe levels of anxiety.

###### Insomnia Severity

Insomnia severity was measured using the Insomnia Severity Index [111], which measures the severity of nighttime and daytime components of insomnia. It consists of 7 items (eg, “How satisfied/dissatisfied are you with your current sleep pattern?”), each rated from 0 (no problem or very satisfied) to 4 (very severe problem or very dissatisfied). The internal consistency is reported to be high (Cronbach α=0.90; 0.86 in this sample). A total score of ≥15 indicates moderate to severe levels of insomnia.

##### Work-Related Outcomes

###### Emotional Exhaustion

Emotional exhaustion was assessed using the emotional exhaustion subscale of the Maslach Burnout Inventory [112], which consists of 5 items (eg, “I feel burned out from my work”) with response options ranging from 1 (never) to 6 (very often). Internal consistency for the emotional exhaustion subscale is high (Cronbach α=0.74 previously; 0.81 in this sample).

###### Work-Related Rumination

Work-related rumination was measured using the 3-item (eg, “Even at home I often think of my problems at work”) subscale of the Irritation Scale [113], with response options ranging from 1 (strongly disagree) to 7 (strongly agree). The subscale shows good internal consistency, with a Cronbach α=0.87 (0.88 in this sample).

###### Work-Related Anxiety

Anxiety related to the induction program was assessed using a self-developed questionnaire consisting of 8 items (eg, “When I think about the *Referendariat*, I notice how everything in me tenses up”), with response options ranging from 0 (does not apply at all) to 4 (applies fully). Internal consistency, measured using the Cronbach α, was high (Cronbach α=0.81).

###### Job Satisfaction

Job satisfaction was measured using an adapted subscale of the Job Diagnostic Survey [114,115], which consists of 4 items (eg, “If I could choose again, I would immediately become a teacher again”) with scores ranging from 1 (does not apply) to 4 (does apply; Cronbach α=0.85; 0.86 in this study).

###### Work-Related Effort and Rewards

Effort and reward were assessed using the Effort-Reward Imbalance questionnaire [116], with response options ranging from 1 (strongly agree) to 4 (strongly disagree). The effort subscale consists of 3 items (eg, “I have many interruptions and disturbances while performing my job”; Cronbach α=0.74; 0.4 in this sample). The reward subscale consists of 7 items (eg, “Considering all my efforts and achievements, my salary/income is adequate”; Cronbach α=0.79; 0.69 in this sample). In addition to analyzing the subscales separately, an effort-reward imbalance ratio was calculated according to the following formula—effort-reward imbalance = effort/(reward × *c*) [117]—with the factor *c* serving as a correction factor for the different number of items in the 2 subscales, corresponding to a value of 0.429. Any value of >1 was theorized to represent an imbalance between efforts invested and rewards received in return [117], whereas any value of >0.715 was empirically derived as the optimal cutoff to identify occupational imbalance situations [118].

###### Absenteeism and Presenteeism

Days of absenteeism and presenteeism over the previous 3 months (since the beginning of the study or the previous 8 weeks for T2) were assessed using pertinent items extracted from the German version of the Trimbos and Institute for Medical Technology Assessment questionnaire on health care consumption and productivity loss in patients with a psychiatric disorder [119].

##### Coping Orientation and Self-Efficacy

###### Problem-Solving Ability

In line with previous research on mechanisms of change in internet-based interventions [120,121], participants’ problem-solving ability was measured as a proxy for problem-focused coping using the revised version of the Social Problem-Solving Inventory (SPSI-R) [122,123]. The SPSI-R was developed by the same group of authors [124] who initially created problem-solving interventions to capture the specific aspects addressed in these types of interventions. Aligned with our intervention’s core concept, problem-solving ability was measured using the 2 subscales of *negative problem orientation* and *avoidance style* from the SPSI-R. A summation score was calculated by adding the 2 subscales (Cronbach α=0.84 in this study). The composite scale consisted of 10 items rated from 0 (does not apply to me at all) to 4 (applies to me very much). Negative problem orientation describes negative appraisal of problems and problem-solving (eg, “I feel threatened and anxious when I have an important problem to solve”). Avoidance style assesses someone’s tendency to avoid solving problems (eg, “I put off solving problems until it is too late to do anything about them”). Low values represent high problem-solving ability, whereas high values indicate a relative lack of problem-solving ability.

###### Emotion Regulation Skills

To measure change in the tendency to use adaptive emotion regulation skills, the Emotion Regulation Skills Questionnaire (ERSQ) was used [125]. Both the emotion regulation part of the intervention and the ERSQ were developed using the same theoretical background [126]; consequently, the ERSQ closely reflects the emotion regulation skills taught in the iSMI. The ERSQ was also previously used to measure change in emotion regulation skills evaluating a similar intervention [98]. In line with the emotion-focused skills taught in the iSMI, the subscales used were *understanding* (eg, “I was aware of why I felt the way I felt”), *acceptance of one’s emotions* (eg, “I accepted my emotions”), and *emotional self-support* when facing emotions (eg, “I supported myself in emotionally distressing situations”). According to the ERSQ manual [125], the summation score was calculated by adding the 3 subscales (Cronbach α=0.82 in this study). Thereby, the combined scale consists of 9 items that are rated from 0 (not at all) to 4 (almost always). Emotion regulation skills are measured as a proxy for emotion-focused coping.

###### CRM Self-Efficacy

To assess CRM self-efficacy, the corresponding subscale of the Ohio State Teacher Efficacy Scale [115,127] was used. This subscale has 8 items (eg, “How much can you do to control disruptive behavior in the classroom?” and “How well can you respond to defiant students?”) that are rated from 1 (very bad or very little) to 6 (very good or very much). Internal consistencies range between 0.84 and 0.92 in the German sample in the study by Kunter et al [115] (Cronbach α=0.86 in this study).

###### Work-Related Self-Efficacy

To assess more general work-related self-efficacy, the Teacher Self-Efficacy Scale was used [128], which consists of 10 items (eg, “I trust myself to inspire the students for new projects”), each rated from 1 (is not true) to 4 (is exactly true; Cronbach α=0.76 to 0.82; 0.78 in this study).

##### Client Satisfaction

User satisfaction with the intervention was measured using the Client Satisfaction Questionnaire [129] adapted to and validated for internet-based interventions (CSQ-I) [130]. The CSQ-I consists of 8 items (eg, “The training has met my needs”), each having response options ranging from 11 to 4, with higher values indicating greater satisfaction. Reliability is high, as indicated by a McDonald ω ranging from 0.93 to 0.95 (Cronbach α in this study’s IG at T2=0.93).

##### Statistical Analyses

###### Overview

In adherence to the CONSORT guidelines [101], data were analyzed on an intention-to-treat (ITT) basis including all participants who were randomized regardless of the amount of the program (if any) they completed. Additional per-protocol sensitivity analyses were conducted for the primary outcome. A 2-tailed significance level of *P≤*.05 was used for all inferential tests.

###### Missing Data

As recommended by Schafer and Graham [131], missing data were estimated using multiple imputations, with 20 estimates calculated for each missing piece of data. The imputed datasets were analyzed separately, and the parameter estimates and hypothesis tests were ultimately pooled. Existing data on the primary and secondary outcomes, as well as the grouping variable and sociodemographic variables, were used in the imputation model.

###### Intervention Effect

To analyze between-group differences immediately after the intervention (T2) and at the 3-month follow-up, analyses of covariance (ANCOVAs) were used, with the corresponding baseline values of each particular outcome used as covariates. In a simulation study comparing different approaches in the analysis of pretest-posttest data in clinical settings, ANCOVAs with baseline scores as covariates were shown to be the most optimal [132]. Between-group Cohen *d* values were calculated for T2 and the 3-month follow-up using the R *esc* package (R Foundation for Statistical Computing) based on *F* values drawn from the pooled ANCOVAs of the multiply imputed datasets. To assess long-term efficacy at the 6-month follow-up, within-subject comparisons were used. For this purpose, repeated-measure ANOVAs were conducted comparing T1 and the 6-month follow-up for each outcome variable. Within-group Cohen *d* values were calculated by controlling for correlations within samples.

###### Response Analysis

For the primary outcome, reliable change and corresponding numbers needed to treat (NNTs) were calculated at T2 and the 3-month follow-up to estimate the practical relevance of the effects [133]. To calculate reliable change, the reliable change index reported by Heber et al [95] was used. These authors calculated the reliable change index based on the norm populations’ SD (6.2) and a reliability of the PSS-10 scale of 0.91 [134]. Thus, participants were defined as having “reliably deteriorated” if their PSS-10 score increased by >5.16 points from T1 to T2 or from T1 to the 3-month follow-up and as having “reliably improved” if their PSS-10 score decreased by >5.16 points from T1 to T2 or from T1 to the 3-month follow-up. On the basis of reliably changed participants, the corresponding NNTs were calculated. The NNT is an epidemiological effect size that indicates the number of participants who must be given access to an intervention to generate 1 additional reliably changed participant compared to the control condition. The NNT is used to communicate treatment effects more comprehensively [135].

###### Sensitivity Analysis

In addition to the preregistered analyses of the ITT sample, per-protocol analyses were conducted for the primary outcome at T2 and the 3-month follow-up to assess the robustness of the results obtained through the ITT analyses. To estimate an intervention’s full potential, only those individuals in the IG who followed the intervention’s protocol adequately were included in the per-protocol analyses. Participants who completed ≥5 of the 7 sessions of the iSMI were considered to have followed the protocol adequately.

###### Mediation Analysis

To examine the hypothesized mediating role of improved problem-focused and emotion-focused coping on the intervention’s effect on perceived stress (primary outcome), a parallel multiple mediation analysis was conducted. The change in mediators from T1 to T2, as well as the 3-month follow-up score of the outcome, was used to establish temporal precedence [136]. Following the recommendations by Hayes and Rockwood [137], the baseline score for the outcome variable was included as a covariate.

###### Ethical Considerations

The Ethics Committee of the Leuphana University of Lüneburg, Germany, approved this study, the trial’s design, and its analysis (EB-Antrag_201712-12). This study was registered at the German Clinical Trials Register (reference DRKS00013880), which is the primary registry recognized by the World Health Organization in Germany. Interested participants were informed about the study, the processing and handling of their data, and that the study data would be deidentified. All participants provided informed consent. Participants received no compensation.

## Results

### Participants and Baseline Characteristics

**Figure 1:**
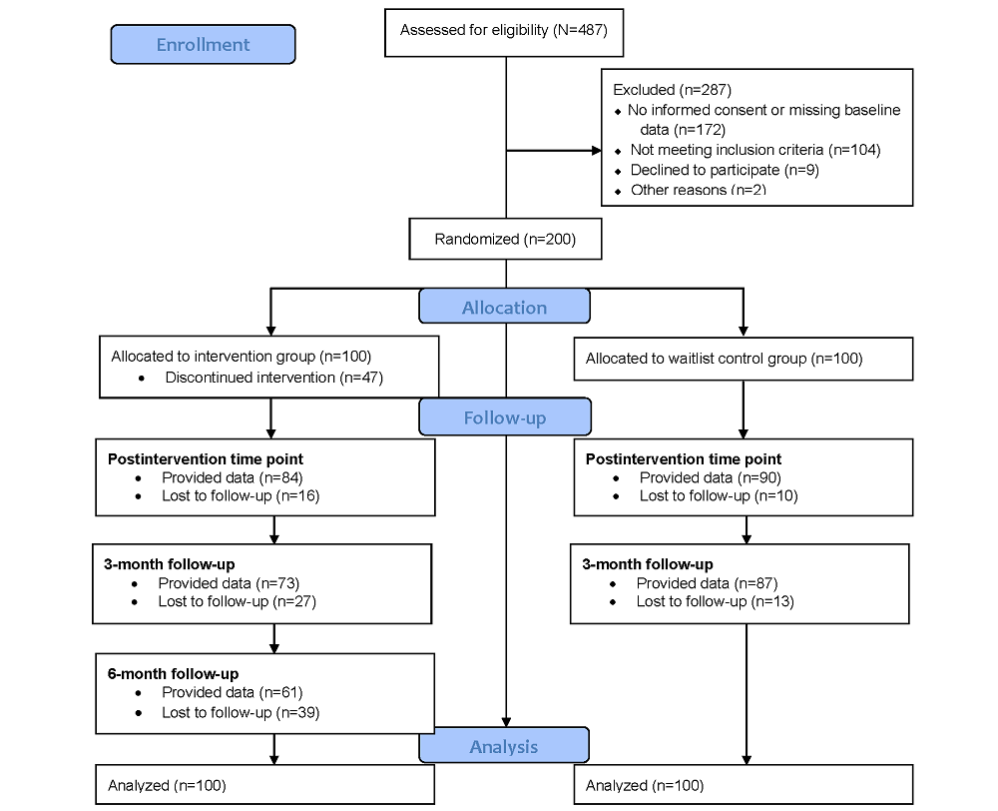
Flow of participants. The 6-month follow-up was only assessed in the intervention group.

Participant flow through the study is shown in Figure 1. Of the 487 individuals who applied for participation (first application: August 7, 2018; last application: May 7, 2019), 287 (58.9%) were excluded. Of these 287 individuals, 172 (59.9%) were excluded for not completing the screening or baseline questionnaire, 104 (36.2%) were deemed ineligible (primarily due to a PSS-10 score below the threshold of >21), 9 (3.1%) declined to participate, and 2 (0.7%) were excluded for other reasons. As a result, 200 participants were randomized into either the IG (n=100, 50%) or the WLG (n=100, 50%).

Table 2 summarizes the baseline characteristics of the sample. Participants were, on average, aged 29 (SD 4.7) years and predominantly female (171/200, 85.5%). Most either had a partner or were married (140/200, 70%), and 80% (160/200) did not have children. At baseline, they had been in the induction phase for an average of 7.4 (SD 4.8) months. The median number of independently taught lessons was 10 per week, with a median class size of 25 (20-27) and a median of 4 (3-6) students rated as challenging (range 0-20). Participants mostly taught at a grammar school (72/200, 36%), followed by a primary school (46/200, 23%). The most frequently taught subjects were German (76/200, 38%), math (53/200, 26.5%), and English (37/200, 18.5%). Roughly three-quarters of the participants at baseline (148/200, 74%) reported an imbalance between effort invested and rewards received in return (Table S2 in Multimedia Appendix 1) based on a theoretically derived cutoff of >1.00 [117]. On the basis of an empirically derived cutoff of >0.715 [118], nearly all (196/200, 98%) reported an occupational imbalance risk situation at baseline. Moreover, the sample was highly distressed, with most reporting clinical symptoms of depression (118/200, 59%) or moderate or severe symptoms of anxiety (118/200, 59%) and more than one-third (77/200, 38.5%) reporting clinical symptoms of insomnia. Most (152/200, 76%) claimed that they had been working over the last 3 months despite feeling unwell. Most reported that they had no experience with any health training program (188/200, 94%), had not consulted a physician for the same problems for which they were attending this training program (189/200, 94.5%), and had not received any psychotherapy in the past (164/200, 82%). Also at baseline, a minority (29/164, 17.7%) said that they had thought about undergoing psychotherapy. The most frequently cited reason for not doing so was concern about having problems when later applying to be a civil servant (14/29, 48%). Tables S3 and S4 in Multimedia Appendix 1 provide a more detailed description of the sample.

**Table 2:** Demographic characteristics of the randomized controlled trial sample consisting of German beginning teachers with increased stress (N=200).

		Total	IG^a^ (n=100)	WLG^b^ (n=100)
Age (y), mean (SD)		29.4 (4.7)	29.4 (4.8)	29.4 (4.6)
**Sex, n (%)**				
	Male	29 (14.5)	13 (13)	16 (16)
	Female	171 (85.5)	87 (87)	84 (84)
**Relationship status, n (%)**				
	Single	58 (29)	25 (25)	33 (33)
	In partnership	93 (46.5)	52 (52)	41 (41)
	Married	47 (23.5)	21 (21)	26 (26)
	Divorced or separated	2 (1)	2 (2)	0 (0)
**Children, n (%)**				
	Yes	40 (20)	20 (20)	20 (20)
	No	160 (80)	80 (80)	80 (80)
**Type of school, n (%)**				
	Primary school	46 (23)	25 (25)	21 (21)
	Grammar school	72 (36)	31 (31)	41 (41)
	Comprehensive school	20 (10)	12 (12)	8 (8)
	Technical school	31 (15.5)	17 (17)	14 (14)
	Special needs school	16 (8)	8 (8)	8 (8)
	Other	15 (7.5)	7 (7)	8 (8)
**Duration of induction phase (mo), n (%)**				
	24	51 (25.5)	24 (24)	27 (27)
	18	149 (74.5)	76 (76)	73 (73)
**Presenteeism in the previous 3 mo, n (%)**				
	Yes	152 (76)	85 (85)	67 (67)
	No	48 (24)	15 (15)	33 (33)
**Absenteeism in the previous 3 mo, n (%)**				
	Yes	92 (46)	50 (50)	42 (42)
	No	108 (54)	50 (50)	58 (58)
Class size, median (IQR)		25 (20-27)	25 (20-26)	25 (21-27)
Challenging students, median (IQR)		4 (3-6)	4 (3-7)	4 (3-5)
Independently taught lessons, median (IQR)		10 (8-12)	10 (8-12)	10 (8-12)
**Experience with health training, n (%)**				
	Yes	12 (6)	7 (7)	5 (5)
	No	188 (94)	93 (93)	95 (95)
**Consulted a physician for the same problems, n (%)**				
	Yes	11 (5.5)	9 (9)	2 (2)
	No	189 (94.5)	91 (91)	98 (98)
**Psychotherapy in the past, n (%)**				
	Yes	36 (18)	24 (24)	12 (12)
	No	164 (82)	76 (76)	88 (88)
Time in induction phase (mo), mean (SD)		7.4 (4.8)	8.4 (5.1)	6.5 (4.2)

^a^IG: intervention group.

^b^WLG: waitlist control group.

### Missing Data

Baseline data were available for all participants. Overall, data for the primary outcome were missing for 13% (26/200) of all participants at T2 (IG: 16/100, 16%; WLG: 10/100, 10%), 20% (40/200) of all participants at the 3-month follow-up (IG: 27/100, 27%; WLG: 13/100, 13%), and 39% (39/100) of those in the IG at the 6-month follow-up. The 2 groups (IG and WLG) did not differ in the percentage of missing data at T2 (χ^2^_1_=1.1; *P=*.29) but did differ at the 3-month follow-up (χ^2^_1_=5.3; *P=*.02). A multivariate ANOVA indicated that there was no meaningful difference in baseline outcomes of participants with versus without missing data at the 3-month follow-up (*F*_1, 13_=1.09; *P=*.37).

### Intervention Use and Client Satisfaction

#### Overview

At T2, a total of 72% (72/100) of the participants responded to the CSQ-I measuring their satisfaction with the entire training program on a 4-point Likert scale (Table S5 in Multimedia Appendix 1). Overall satisfaction with the program was high (mean 27.31, SD 4.31). A clear majority (46/72, 64%) of the respondents stated that they would definitely recommend the training program to a friend in need, whereas 26% (19/72) said that they would somewhat recommend it, and the remaining 10% (7/72) said that they either would not or would somewhat not recommend it. Most felt that the training had helped them deal with their problems more effectively (32/72, 44% strongly agreed, whereas 32/72, 44% somewhat agreed). Meanwhile, 10% (7/72) somewhat did not agree, and 1% (1/72) did not agree.

Regarding intervention use, of the 100 individuals randomized to the IG, 6 (6%) did not log into the training platform at all, whereas 9 (9%) logged into the training platform but did not complete even 1 session.

#### SMI Completion

The proportions of participants who completed each session of the iSMI were 85% (85/100) for session 1, a total of 71% (71/100) for session 2, a total of 57% (57/100) for session 3, a total of 52% (52/100) for session 4, a total of 50% (50/100) for session 5, and 46% (46/100) for session 6. The entire intervention, with its 7 sessions, was completed by 38% (38/100) of the users. Reasons for dropout were reported by 17% (17/100) of the users. The most common reasons reported were lack of time (12/17, 71%), lack of motivation (10/17, 59%), and lack of personal contact (6/17, 35%). On average, users completed 4 (SD 2.9) of the 7 sessions. This corresponds to 57% (4/7) of the overall intervention. The median words written by the participants in the training program were 1314 (IQR 326-3121).

#### Optional CRM Skill Module

At least one session of the CRM module was completed by 57% (57/100) of the participants in the IG, at least 2 sessions were completed by 47% (47/100), at least 3 sessions were completed by 30% (30/100), at least 4 sessions were completed by 19% (19/100), at least 5 sessions were completed by 13% (13/100), at least 6 sessions were completed by 11% (11/100), and all 7 sessions were completed by 6% (6/100) of the participants. Participants averaged writing 267 (SD 504) words, with a median of 46 words and a word IQR from 7 to 250 words. Table S6 in Multimedia Appendix 1 provides an overview of the rated helpfulness and rated usefulness of the sessions used.

#### Postintervention Transfer of Training Elements Into Daily Life

At the 3-month follow-up, participants were asked to what extent they were able to use the different strategies learned in their daily life after the training program. They most often listed making use of positive activities, with 40% (23/58) reporting using them nearly daily, 40% (23/58) reporting using them often, 14% (8/58) reporting using them sometimes, and 5% (3/58) reporting using them rarely. More than half (32/58, 55%) of the respondents in the IG indicated almost daily use of at least one strategy, whereas a large majority (47/58, 81%) indicated frequent use of at least one strategy. Pertaining to strategies taught within the teacher-specific individualized modules, 9% (5/58) indicated using those almost daily, 29% (17/58) indicated using them often, 16% (9/58) indicated using them sometimes, and 14% (8/58) indicated using them rarely. Table S7 in Multimedia Appendix 1 provides a more detailed description of the results.

### Use of Care as Usual

Participants reporting having sought additional help (eg, other health training, general practitioner, or psychotherapist) due to their stress levels since the start of the study did not differ significantly between the 2 study groups at T2 (IG: 10/64, 16%; WLG: 11/70, 16%; *P*=.99) or at the 3-month follow-up (IG: 7/59, 12%; WLG: 10/72, 14%; *P*=.93).

### Intervention Effect

For the intention-to-treat sample, means and SDs for all outcomes at all time points are shown in Table 3.

**Table 3:** Outcomes at baseline (T1), the postintervention time point (T2), 3-month follow-up (3-MFU), and 6-month follow-up (6-MFU)—intention-to-treat sample.

Outcome				T1, mean (SD)					T2^a^, mean (SD)						3-MFU^a^, mean (SD)						6-MFU^a^—IG^b^, mean (SD)
				IG			WLG^c^	IG		WLG			IG			WLG					
**Primary outcome**																					
	Perceived stress			26.5 (4.2)		25.7 (4.4)			20.4 (6.8)			23.5 (6.0)			18.7 (7.7)			22.6 (6.1)			17.1 (7.3)
**Secondary outcomes**																					
	**Mental health**																				
		Depression	20.8 (8.3)		19.0 (8.5)			14.2 (7.8)			19.2 (9.0)			14.2 (9.1)			18.1 (8.9)			13.3 (8.9)	
		Anxiety	11.4 (4.3)		11.2 (4.8)			8.0 (4.4)			10.8 (5.0)			7.6 (4.5)			10.4 (5.2)			6.6 (5.2)	
		Insomnia	12.9 (6.0)		11.4 (6.0)			10.1 (5.6)			11.8 (6.3)			9.4 (5.5)			10.9 (6.1)			7.3 (4.8)	
	**Work-related outcomes**																				
		Emotional exhaustion	23.4 (4.2)		22.9 (4.4)			21.1 (5.3)			23.0 (5.1)			20.4 (5.5)			22.5 (4.8)			18.6 (5.3)	
		Work-related rumination	17.7 (3.6)		17.9 (3.1)			15.7 (4.5)			18.1 (3.0)			15.0 (4.4)			17.0 (3.5)			12.4 (4.8)	
		Work-related anxiety	20.6 (6.0)		19.0 (6.5)			15.3 (7.4)			18.2 (7.0)			15.9 (8.5)			17.9 (6.9)			14.4 (10.2)	
		Job satisfaction	10.6 (3.2)		10.9 (3.0)			11.4 (3.3)			10.4 (3.1)			11.4 (3.2)			10.7 (3.1)			11.8 (3.4)	
		Effort	9.4 (1.6)		9.2 (1.3)			8.6 (1.7)			9.2 (1.6)			8.8 (1.8)			9.1 (1.7)			7.6 (2.0)	
		Reward	17.8 (3.4)		18.4 (3.7)			18.4 (3.4)			17.9 (3.8)			18.3 (3.9)			17.5 (3.7)			18.3 (4.3)	
		Absenteeism days^d,e^	2.7 (5.5)		3.8 (16.0)			2.5 (4.0)			1.8 (4.6)			2.0 (3.1)			2.2 (3.3)			3.7 (16.5)	
		Presenteeism days^d,e^	12.3 (15.9)		7.5 (11.5)			10.7 (15.7)			9.5 (15.7)			9.8 (17.9)			9.4 (15.7)			5.4 (13.0)	
	**Coping orientation and self-efficacy**																				
		Emotion regulation skills	19.9 (6.0)		19.3 (4.8)			24.8 (5.3)			21.0 (5.1)			25.7 (5.6)			21.0 (5.6)			26.0 (5.2)	
		Problem-solving ability^f^	19.5 (6.1)		18.7 (5.9)			14.5 (6.4)			17.6 (6.8)			14.3 (7.2)			17.5 (7.1)			12.0 (6.4)	
		Classroom management self-efficacy	30.9 (6.0)		30.0 (5.4)			33.5 (5.6)			30.8 (6.0)			34.9 (5.6)			32.1 (6.0)			34.8 (7.1)	
		Work-related self-efficacy	27.5 (4.3)		26.9 (10.9)			30.0 (4.4)			27.6 (4.2)			31.6 (3.9)			27.9 (4.7)			30.4 (5.0)	

^a^Missing values were imputed via multiple imputations. The 6-MFU was only assessed in the intervention group.

^b^IG: intervention group.

^c^WLG: waitlist control group.

^d^Missing values were not imputed for absenteeism or presenteeism days.

^e^Absenteeism and presenteeism days during the previous 3 months for T1, the 3-MFU, and the 6-MFU and since the beginning of the study (ie, previous 8 weeks) for T2.

^f^High values represent negative problem orientation and high avoidance of problems and, thus, poor problem-solving ability.

#### Primary Outcome Analyses

#### Overview

At T2, individuals in the IG reported significantly less perceived stress than waitlist controls (*F*_1, 145_*=*13.6; *P<*.001), with a mean PSS-10 score 3.1 points lower than that of the WLG. The standardized mean difference was *d=*0.52 (95% CI 0.24-0.80). Perceived stress from T1 to T2 decreased by an average of –6.2 (7.63) points in the IG versus –2.2 (5.53) points in the WLG.

**Figure 2:**
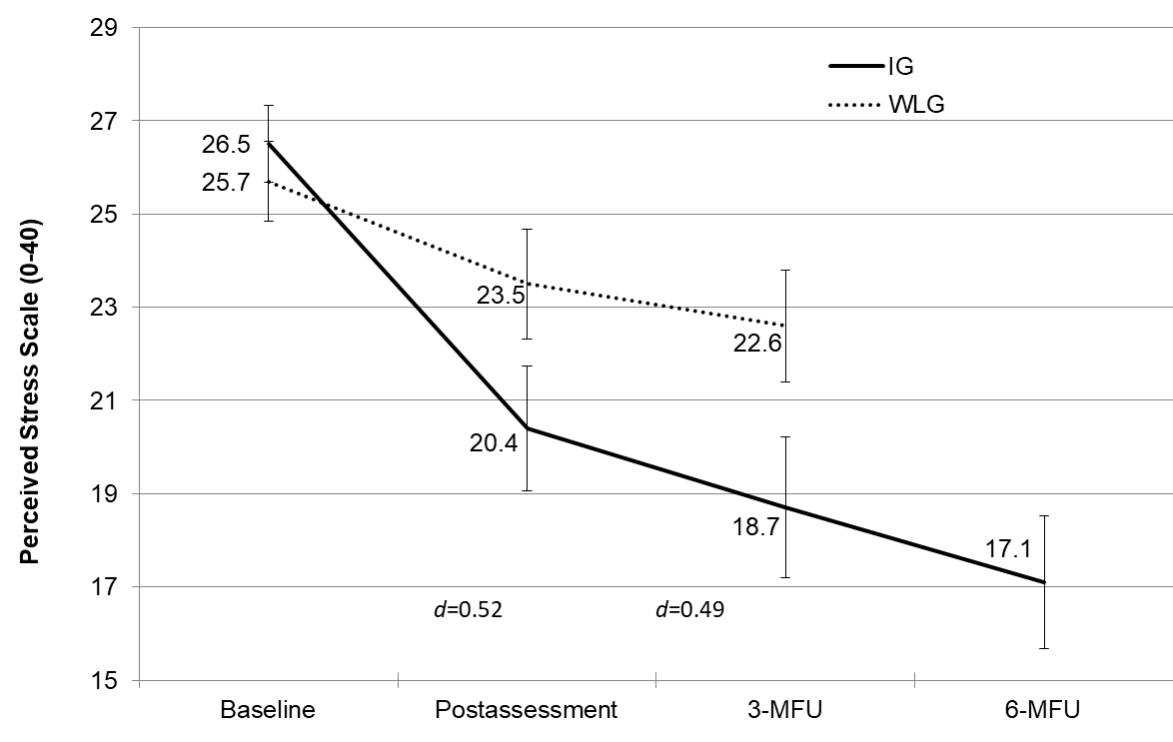
Means with 95% CIs on the primary outcome measure of perceived stress (intention to treat; N=200) measured at baseline, the postintervention time point, the 3-month follow-up (3-MFU), and the 6-month follow-up (6-MFU) in the intervention group (IG) and waitlist control group (WLG).

At the 3-month follow-up, individuals in the IG also reported significantly less perceived stress than those in the WLG (*F*_1, 76_*=*12.0; *P=*.001), with the IG’s mean being 3.9 (0.94) points lower. The standardized mean difference was 0.49 (95% CI 0.21-0.77). From the 3-month follow-up to the 6-month follow-up, the level of perceived stress in the IG decreased further. A within-subject ANOVA from T1 to the 6-month follow-up showed a significant effect (*F*_1, 43_*=*76.9; *P<*.001). Moreover, the within-subject effect size was large (∆ of the 6-month follow-up – T1=–9.4; *d=*1.57, 95% CI 1.16-1.95). Table 4 summarizes the intervention effects for the primary and secondary outcomes at T2 and the 3-month follow-up. Table S8 in Multimedia Appendix 1 shows the within-subject ANOVAs from T1 to the 6-month follow-up in the IG. Figure 2 shows the progression of PSS-10 scores for both groups from baseline to the 6-month follow-up.

**Table 4:** Results of analyses of covariance and Cohen *d* values for the primary and secondary outcomes at the postintervention time point (T2) and 3-month follow-up (3-MFU)—intention-to-treat sample.

Outcome					Differences between intervention group and waitlist control group																
					T2										3-MFU						
					*F* test (*df*)			*P* value			Cohen *d* (95% CI)			*F* test (*df*)				*P* value			Cohen *d* (95% CI)
**Primary outcome**																					
	Perceived stress			13.6 (1, 145)			<.001			0.52 (0.24 to 0.80)			12.0 (1, 76)				<.001			0.49 (0.21 to 0.77)	
**Secondary outcomes**																					
	**Mental health**																				
		Depression	21.8 (1, 77)			<.001			0.66 (0.38 to 0.94)			11.2 (1, 92)				<.001			0.47 (0.19 to 0.75)		
		Anxiety	23.3 (1, 98)			<.001			0.68 (0.40 to 0.97)			16.3 (1, 85)				<.001			0.57 (0.29 to 0.85)		
		Insomnia	18.0 (1, 166)			<.001			0.60 (0.32 to 0.88)			5.7 (1, 55)				.02			0.34 (0.06 to 0.62)		
	**Work-related health**																				
		Emotional exhaustion	12.1 (1, 82)			<.001			0.49 (0.21 to 0.77)			8.4 (1, 57)				.006			0.41 (0.13 to 0.69)		
		Work-related rumination	18.3 (1, 90)			<.001			0.60 (0.32 to 0.89)			10.3 (1, 67)				.002			0.45 (0.17 to 0.74)		
		Work-related anxiety	23.3 (1, 158)			<.001			0.68 (0.40 to 0.97)			7.4 (1, 71)				.008			0.38 (0.10 to 0.66)		
		Job satisfaction	14.9 (1, 147)			<.001			0.55 (0.26 to 0.83)			8.8 (1, 107)				.004			0.42 (0.14 to 0.70)		
		Effort	8.6 (1, 87)			.004			0.42 (0.14 to 0.70)			2.3 (1, 63)				.14			0.21 (–0.07 to 0.49)		
		Reward	3.2 (1, 113)			.08			0.25 (–0.03 to 0.53)			4.4 (1, 73)				.04			0.30 (0.02 to 0.57)		
		Absenteeism days^a,b^	1.5 (1, 152)			.22			0.20 (–0.12 to 0.51)			0.15 (1, 128)				.70			0.07 (–0.28 to 0.41)		
		Presenteeism days^a,b^	0.06 (1, 152)			.80			0.04 (–0.27 to 0.36)			0.54 (1, 112)				.46			0.13 (–0.22 to 0.47)		
	**Coping and self-efficacy**																				
		Emotion regulation skills	23.8 (1, 130)			<.001			0.69 (0.40 to 0.98)			26.0 (1, 88)				<.001			0.72 (0.43 to 1.01)		
		Problem-solving ability	16.5 (1, 152)			<.001			0.57 (0.29 to 0.86)			12.3 (1, 93)				<.001			0.50 (0.21 to 0.78)		
		CRM^c^ self-efficacy	8.0 (1, 115)			.006			0.40 (0.12 to 0.68)			7.6 (1, 83)				.007			0.39 (0.11 to 0.67)		
		Work-related self-efficacy	14.1 (1, 89)			<.001			0.53 (0.25 to 0.81)			24.3 (1, 55)				<.001			0.69 (0.41 to 0.98)		

^a^Missing values were not imputed for absenteeism or presenteeism days.

^b^Absenteeism and presenteeism days during the previous 3 months for T1 and the 3-MFU and since the beginning of the study (ie, previous 8 weeks) for T2.

^c^CRM: classroom management.

#### Response Analysis

At T2, a total of 50% (50/100) of the participants in the IG reported reliable improvement versus just 23% (23/100) in the WLG. Meanwhile, 5% (5/100) in the IG reported reliable deterioration versus 9% (9/100) in the WLG, and 45% (45/100) and 68% (68/100) in the IG and WLG reported no reliable change, respectively.

At the 3-month follow-up, 61% (61/100) in the IG and 33% (33/100) in the WLG reported reliable improvement, whereas 4% (4/100) in the IG and 6% (6/100) in the WLG reported reliable deterioration, and 35% (35/100) in the IG and 60% (60/100) in the WLG reported no reliable change. This corresponds to an NNT of 3.70 (95% CI 2.51-7.05) at T2 and 3.70 (95% CI 2.48-7.30) at the 3-month follow-up. Thus, to observe 1 participant with a reliable improvement as compared to the waitlist group, access to the intervention needs to be given to 4 individuals. At the 6-month follow-up, 67% (67/100), 6% (6/100), and 27% (27/100) of the participants in the IG reported reliable improvement, reliable deterioration, and no reliable change, respectively.

#### Sensitivity Analysis

Analyzing only IG participants who adhered to the protocol and finished at least 5 sessions (50/100, 50% of the participants), the ANCOVA at T2 revealed a significant difference relative to the WLG (*F*_1, 141_*=*40.5; *P<*.001), with a between-group effect considered large (∆ IG_T2_ – WLG_T2_ = –6.0 points on the PSS-10; *d=*1.1, 95% CI 0.74-1.46).

#### Dose-Response Analysis

In a simple regression model, the number of sessions completed significantly predicted reduction in the PSS-10 score from T1 to T2 (*b*=–1.2; *P<*.001), indicating that each session completed led to an average reduction of 1.2 (2.74) points on the PSS-10. This indicates a statistically significant dose-response relationship.

#### Secondary Outcome Analyses

##### Mental Health Outcomes

As displayed in Table 4, at T2 and the 3-month follow-up, individuals in the IG reported significantly fewer symptoms of depression, anxiety, and insomnia than participants in the WLG. At T2, effect sizes were moderate to large and ranged from *d=*0.60 for insomnia severity to *d=*0.68 for anxiety symptoms. At the 3-month follow-up, effect sizes were small to moderate and ranged from 0.34 for insomnia severity to 0.57 for anxiety symptoms. Within-subject effect sizes at the 6-month follow-up were large, ranging from *d=*0.87 for depression to *d=*1.01 for insomnia severity.

##### Work-Related Outcomes

With regard to work-related outcomes, individuals in the IG reported significantly less emotional exhaustion (T2: *d=*0.49; 3-month follow-up: *d=*0.41), work-related rumination (T2: *d=*0.60; 3-month follow-up: *d=*0.45), and work-related anxiety (T2: *d=*0.68; 3-month follow-up: *d=*0.38) at T2 and the 3-month follow-up. From the 3- to the 6-month follow-up, scores further decreased in the IG, and within-subject effect sizes were large, ranging from *d=*0.70 for work-related anxiety to *d=*1.23 for work-related rumination at the 6-month follow-up. Job satisfaction improved significantly among participants in the IG at T2 (*d=*0.55) and the 3-month follow-up (*d=*0.42). The within-subject difference between T1 and the 6-month follow-up was significant, with an effect size of *d=*0.36. The IG reported significantly reduced effort invested at T2 but not at the 3-month follow-up. For rewards received in return, the pattern observed was opposite to this, with significantly increased rewards received in return at the 3-month follow-up in the IG but insignificant findings at T2. No significant between-group differences were found for absenteeism or presenteeism days at either T2 or the 3-month follow-up.

##### Coping Orientation and Self-Efficacy

Participants in the IG showed significantly reduced avoidance and negative problem orientation, indicating improved problem-solving ability of moderate size relative to the WLG at T2 (*d=*0.57) and the 3-month follow-up (*d=*0.50). Participants in the IG also reported significantly improved emotion regulation skills of moderate effect size at T2 (*d=*0.69) and of moderate to large effect size at the 3-month follow-up (*d=*0.72) on the ERSQ. Teachers’ general self-efficacy and CRM self-efficacy were significantly higher in the IG at T2 and at the 3-month follow-up than in the WLG. The effect on general teacher self-efficacy can be considered moderate to large (T2: *d*=0.53; 3-month follow-up: *d*=0.69), whereas the effect was small to moderate for CRM self-efficacy (T2: *d=*0.40; 3-month follow-up: *d=*0.39). Within-subject analyses comparing T1 and the 6-month follow-up showed significant effects of moderate to large size. Within-subject effect sizes ranged from *d=*0.58 for CRM self-efficacy to *d=*1.20 for problem-solving ability. Within a per-protocol analysis of the CRM module including only those participants who engaged with it more intensively (24/100, 24% who wrote at least the average of 267 words), the between-group effect sizes were slightly larger. For CRM self-efficacy at T2, the between-group effect was moderate to large (∆ IG_T2_ – WLG_T2_ = 2.8; *d*=0.62, 95% CI 0.17-1.08), whereas for general teacher-specific self-efficacy, it was large (*d*=0.91, 95% CI 0.45-1.37).

### Mediation Analysis

**Figure 3:**
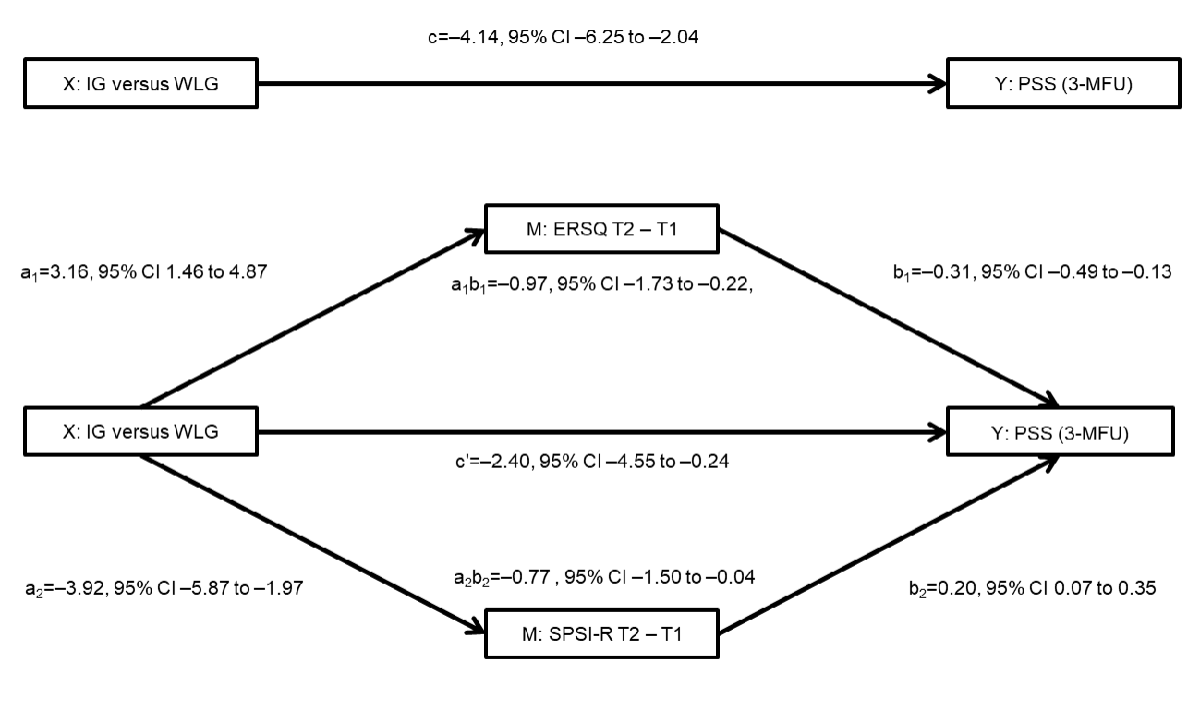
Parallel multiple mediation model with 3-month follow-up (3-MFU) perceived stress scores as the outcome variable, change in emotion regulation skills (Emotion Regulation Skills Questionnaire [ERSQ]) and problem-solving ability (Social Problem-Solving Inventory–Revised [SPSI-R]) as mediators, and baseline values of perceived stress as covariates. Treatment is coded as 0=waitlist control group (WLG) and 1=intervention group (IG). Path diagrams represent statistically significant mediation effects. Unstandardized β coefficients are shown with 95% CIs. a1: path from the independent variable to the first mediator (ERSQ); a2: path from the independent variable to the second mediator (SPSI-R); b1: path from the first mediator (ERSQ) to the dependent variable (perceived stress); b2: path from the second mediator (SPSI-R) to the dependent variable (perceived stress); M: mediator; PSS: Perceived Stress Scale; T1: baseline; T2: postintervention time point; X: independent variable; Y: dependent variable (perceived stress).

Parallel multiple mediation analysis showed that improvements from T1 to T2 in problem-solving ability (indirect path a_2_b_2_=–0.77, 95% CI –1.50 to –0.04), as well as improvements in emotion regulation skills (indirect path a_1_b_1_=–0.97, 95% CI –1.73 to –0.22), significantly mediated the effect of the intervention on stress levels at the 3-month follow-up. The direct effect of the intervention in terms of reducing stress remained significant at the 3-month follow-up after the mediators were incorporated into the model (c′=–2.40, 95% CI –4.55 to –0.24; Figure 3).

## Discussion

### Principal Findings

This study had 2 objectives: to evaluate the efficacy of an iSMI tailored for beginning teachers and examine the underlying mechanisms of change. The results suggest that this tailored and guided iSMI is effective at reducing perceived stress in beginning teachers. Furthermore, there was evidence that problem-solving ability and emotion regulation skills are mechanisms that work in concert to produce a beneficial effect on stress, as predicted by the transactional stress theory [84].

Addressing the study’s first aim, the results suggest an even larger effect than what was expected a priori on perceived stress in beginning teachers immediately after the intervention. The difference between the IG and WLG was 3.1 points on the PSS-10, which is an incremental reduction of 13.2%. This difference exceeded the threshold for practically meaningful effects [103]; similarly, the reduction can be considered a practically important benefit in a nonclinical sample [138]. This beneficial effect was still evident at the 3- and 6-month follow-up assessments. Significant effects at all assessment points were also identified for various secondary mental health–related outcomes, including depression, anxiety, and insomnia, as well as for most work-related health outcomes, including emotional exhaustion, work-related rumination, work-related anxiety, and job satisfaction. However, they were only partly significant with regard to perceived effort invested and rewards received in return and nonsignificant for presenteeism and absenteeism.

The results of this study for the primary outcome—perceived stress—will be discussed in the following paragraphs, first in light of evidence from previous meta-analyses on occupational internet-based interventions and then in light of evidence from trials evaluating a generic version of this intervention conducted in the general working population. Thereafter, results will be discussed in light of studies on teachers’ mental health.

The effect size (*d=*0.52) for perceived stress at the postintervention time point is comparable to the moderate effect sizes found in meta-analyses of occupational internet-based interventions in the general working population (*g*=0.3-0.54) [69-71]. However, it is slightly smaller than the effects found in subgroup analyses of guided iSMIs in the general population (*g*=0.64) [69]; face-to-face SMIs (*g*=0.73) [139]; and individual-focused, predominantly face-to-face occupational health interventions (*g*=0.65) [140].

The efficacy of the same iSMI, though untailored, has been assessed with different forms of guidance in the general working population. The effect size (*d=*0.52) for perceived stress in this study is somewhat smaller than effects ranging from *d=*0.71 to *d=*0.96 in the general working population with guidance [95], as well as with adherence-focused guidance [98,99] and with self-help [96,97,99]. While there was a difference in the PSS-10 score between the IG and WLG of 3.1 points, previously published trials have mostly shown differences of >5 points, which is just about large enough to be of practical importance [103]. There might be several reasons for the attenuation of the effect on the PSS-10 score observed in this study relative to previous studies.

First, the results of this study are in line with those of research that has linked younger age to lower iSMI efficacy. Participants in this study were, on average, approximately 13 years younger than the average samples of previous trials evaluating the same iSMI. In meta-analyses, younger age has been found to be associated with reduced treatment effect [70,141]. Phillips et al [70] found an effect for occupational internet-based interventions in employees aged <40 years of *g=*0.29, whereas Harrer et al [68] found that internet interventions in university students with an average age of 24 years led to stress reductions with an effect size of *g=*0.20. Although this intervention compares favorably to those interventions in younger people, it is an unsolved question why younger age seems to be a limiting factor regarding efficacy.

A second potential reason for the reduced effect that we observed might be reduced adherence to the program as previous meta-analyses indicate associations between lower intervention adherence and reduced efficacy [142] and between younger age and lower adherence [143]. Participants in this study completed 1.7 fewer main sessions than participants in the study by Heber et al [95] despite us expending considerable effort to increase adherence, including guidance [144], web-based exercises [145], tailoring [146], and email reminders [147]. Comparing the per-protocol analyses of all trials that have assessed an iSMI—by only considering those users who achieved a recommended number of training sessions—all previous studies detected almost the same level of effect [95,97-99], with Cohen *d* values ranging from 0.96 to 1.1, similar to our trial’s *d=*1.1.

Third, although research on moderators of the interplay between efficacy and adherence have thus far focused on intervention design (eg, guidance [148] or individual factors such as age [143]), work-related and organizational factors such as the psychosocial safety climate of an organization [149] could also have moderated adherence and efficacy. Unfortunately, these have rarely been considered. In this study, all participants—in addition to facing the common demands of teaching for the first time—went through an 18- to 24-month long transition phase of induction to teaching. This trainee phase is characterized by repeated classroom visits, evaluations, and exams. As all this is time-consuming and positive evaluations are essential for subsequent tenure, the trainee phase is often described as highly demanding [10,11,16]. At the same time, the organizational context of such participation might indicate a relatively poor psychosocial safety climate as the main reason for nonadherence reported by the teachers in this study was lack of time and these teachers could not use the iSMI during their regular work time. Moreover, receiving a diagnosis that reflects psychological distress during the trainee phase might be a reason for a young teacher not to receive tenure as a civil servant, which is further evidence of the poor psychosocial safety climate. Insufficient time and a poor work environment have been suggested as adherence-reducing factors [146,150,151] and discussed as factors hindering both occupational learning [152] and training transfer [153].

Finally, regarding intervention efficacy, our results should be discussed in the context of interventional research on teachers’ mental health. Effects on stress reduction found in meta-analyses have varied from *d=*0.53 for mindfulness-based interventions for in-service teachers [154] to *d=*0.27 for interventions using cognitive behavioral therapy and relaxation and *d=*0.15 for cognitive behavioral therapy only [155]. With regard to burnout, Iancu et al [72] reported an effect of *d=*0.18, whereas Oliveira et al [156] found effects ranging from *g=*0.11 to *g=*0.18 for social and emotional learning interventions. What is noteworthy is that the effects of interventions for teachers seem to be lower than for digital and face-to-face interventions designed for other occupational groups [69-71,140], emphasizing the need for special consideration of the educational sector. The results of this study compare favorably to most teacher-focused interventions [72,155,156] and suggest that an internet intervention for beginning teachers is at least as effective as existing interventions for in-service teachers that are mainly delivered face-to-face.

Significant effects at all assessment points were also found for various mental health–related secondary outcomes, as well as for most work-related health outcomes. The effects on depression and anxiety symptoms compare favorably to meta-analytic findings for iSMIs in general [69] and for internet interventions for employees [157]. The effect of the intervention on insomnia was similar in our sample to the encouraging results reported for internet-based interventions in general employees [70], demonstrating the impact of stress on sleep quality. Considering the importance of mental detachment and low work-related rumination as major preconditions for recovery from occupational stress, the reduction we observed in work-related rumination seems promising and was slightly larger in our study than the results reported for a recent meta-analysis [158].

Finally, no effects were found for absenteeism and presenteeism, a finding that is consistent with meta-analysis results for digital interventions that indicate a small but nonsignificant effect for both [159].

The second primary aim of this study was to investigate changes in problem-solving ability and emotion regulation skills as mediators of change when an SMI is used. According to the intervention’s program theory, participants reported significant improvements that were hypothesized for both mediators (ie, their problem-solving ability and their emotion regulation skills). More specifically, our results support the assumption that beginning teachers were successful at reducing their general negative orientation toward stressors by starting to expose themselves to problems in a systematic manner as part of the problem-solving part of the intervention [121]. Similarly, the increased emotion regulation skills suggest that users of the emotion regulation part of the program learned to react to stressful situations by stepping back for a moment, trying to understand their negative emotional response, accepting this emotional reaction, and initiating actions to support themselves in that situation. To the best of our knowledge, we have, for the first time, demonstrated that, consistent with *transactional stress theory* [84,160], changes from before to after the intervention in both problem-solving ability and emotion regulation skills may have significantly mediated the intervention’s effect on subsequent levels of perceived stress.

Such findings strengthen the rationale for combining elements in SMIs aiming to empower participants to actively change stressors (eg, problem-solving [161]), with elements aimed at helping teachers accept unpleasant feelings that arise from change-resistant stressors [126]. While actively changing stressors follows the tradition of problem-solving approaches [86,161], coping by acceptance is rooted in mindfulness- and acceptance-based interventions [87,88]. To date, only a few studies have investigated emotion regulation skills without assessing problem-solving ability. For example, emotion regulation skills have been found to be a mechanism of change in a simple mediation model on perceived stress [98] and depressive symptoms [162], but active coping strategies were not considered mechanisms of change in these studies. One value of our research is that it empirically suggests that (1) a balanced repertoire of problem-solving abilities and emotion regulation skills can be achieved and (2) improvements in both coping orientations may have mediated the intervention’s effect on stress reduction. These results are consistent with both transactional stress theory and findings that suggest that the context-dependent use of both problem-focused and emotion-focused coping is superior to using either approach on its own [93,94].

On the other hand, another aspect of the intervention’s program theory could not be tested—specifically, that iSMI users improved their ability to differentiate between controllable and noncontrollable stressors as part of the first session and, by doing so, became able to apply more problem-focused coping approaches for controllable situations and more emotion-focused coping strategies for less controllable situations.

### Limitations of This Study

Several limitations of this study must be considered. First, the results for both efficacy and mechanisms of change should be considered in light of the population studied, which comprised mostly female beginning teachers going through a highly demanding transition phase seeking to receive tenure as civil servants at the end of the trainee phase. As the transition or trainee phase differs between countries, generalizing our results to beginning teachers in other countries might be problematic.

Second, the intervention was successful at reducing a major risk factor for mental disorders and depressive and anxiety symptoms. However, it is unclear whether the intervention has the potential to reduce the later incidence of mental disorders when teachers are in service. This said, similar internet-based interventions have already demonstrated that a reduced incidence of mood disorders is achievable in workers [163,164].

Third, as not all participants used the same individualized modules, the intervention was not the same for all. This could be regarded as a limitation to internal validity. However, responding to the personal needs of participants and tailoring interventions requires that interventions be flexible [64,73,74,165]. There is also evidence that some content was not equally helpful to all teachers. For example, teachers of older students reported more difficulties implementing the sessions on setting rules and routines as they felt that this was more suitable for younger students. There may also have been differences in the perceived usefulness of the training program for other content depending on the subject and age group taught by the participants. This needs to be investigated in future research.

Fourth, the study design does not allow us to draw conclusions regarding the specific effects of particular elements of the intervention, nor can it show that tailoring the intervention or adding the CRM modules had an incremental, practically relevant effect. This said, participants who were more engaged with the CRM modules tended to benefit more from the intervention with regard to work-related self-efficacy.

Fifth, although the intervention was designed based on distinguishing between problem- and emotion-focused coping, the measurement instruments used to investigate these 2 mechanisms were not specific coping questionnaires [166]. Problem-solving ability and emotion regulation skills are only proxies for actual coping. Therefore, replication studies are needed that use operationalizations that align better with specific measures of coping.

Sixth, while this research results suggest that improvements in the proxies for dispositional problem- and emotion-focused coping explain the observed effect on stress reduction in general, more specific questions remain unanswered. The goodness-of-fit hypothesis, for example, states that this effect only becomes apparent in people who have learned to use relatively more problem-orientated coping in controllable situations and relatively more emotion-orientated coping in less controllable situations [167]. However, other research suggests that some coping strategies appear to be adaptive regardless of the specific situation, whereas others depend on the characteristics of the situation (eg, controllability [168]). Future intervention research should complement dispositional coping measures with situation-specific measures to take greater account of the possible influence of the context.

Seventh, no precautions were taken against possible contamination bias, and it cannot be ruled out that participants in the WLG received detailed information about the intervention from participants in the IG by mischance. However, any contamination bias would likely have led to reduced stress in the WLG, thereby reducing between-group differences.

### Implications for Future Research and Practice

Our results have several implications for future research and practice. First, one practical implication is that it might be fruitful to adapt SMIs to specific professions and contexts. In addition to this, it might be advantageous to teach more general stress management skills to identify profession- and role-specific stressors and tackle those in individualized skill development modules. User satisfaction results and nonsystematic feedback received from study participants suggest that program users appreciated the program’s consideration of role-specific stressors. Future studies should systematically compare such tailoring to untailored versions in terms of uptake, acceptability, and efficacy.

Second, it is not clear whether the improvement observed in CRM self-efficacy also translates to improved CRM skills as rated by, for example, independent observers or whether the intervention’s effect also positively impacts student outcomes. Future studies should assess whether exposure to the intervention also impacts teacher behaviors in the classroom and student outcomes.

A third important implication of this study is that “it takes two to tango.” In other words, SMIs should include both problem- and emotion-focused coping strategies, an assertion already expressed by Lazarus and Folkman [84]. Interventions that rely solely on problem-focused elements, such as problem-solving, may frustrate users trying to change unchangeable stressors. Similarly, interventions that emphasize acceptance may lead to premature termination of efforts to alter changeable stressors. Future studies should assess the (perceived) controllability of stressors [169] as a moderator of the efficacy of problem-focused interventions, emotion-focused interventions, and interventions that combine these 2 approaches. The controllability of stressors might also explain the considerable heterogeneity found in previously published meta-analyses assessing SMIs [69-71,140].

Fourth, regarding implementation, it is noteworthy that many participants in our study claimed not to have sought professional help for mental health issues before as they were concerned about the potential for negative professional consequences when they later applied for tenure. For this reason, they appreciated that the intervention was provided by a university as an external institution and that they were permitted to take part anonymously.

Fifth, the observed attenuation of the effect might be related to program use not being possible during the teachers’ work time. This is another indicator of the poor psychosocial safety climate that currently exists in the educational sector [149]. Thus, investigating the assertion proposed by Dollard and Bakker [149] that interventions are more successful if they are embedded in organizations with a high psychosocial safety climate seems worthwhile. In addition, future research should assess psychosocial safety climate as a moderating factor that influences the effect of individual-directed occupational interventions [170] or combine individual-directed interventions with organization-directed safety climate interventions [171]. In the educational sector, such measures could help ensure that participation without fear of negative consequences is possible and that time resources are made available.

Sixth, the substantial dose-response relationship we observed could be used to increase users’ intention to use iSMIs by strengthening individual performance expectations and, at the organizational level, by improving facilitating conditions or, in other words, enhancing the psychosocial safety climate.

### Conclusions

In conclusion, this study contributes to the growing body of evidence supporting the effectiveness of iSMIs, demonstrating their efficacy in beginning teachers undergoing a highly demanding transition phase when they are tailored to their personal problems and needs. In our study, beginning teachers participating in the training program reported significantly reduced perceived stress, depression, and anxiety, and these effects were sustained up to 6 months later. This study also suggests the importance of considering organizational context as a potential moderating factor that might impact the efficacy of individual-directed interventions in the educational sector. Furthermore, they suggest that problem- and emotion-focused coping orientations may be mechanisms of change within an iSMI, adding further credence to the transactional stress theory by Lazarus and Folkman [84], which could help guide the development and refinement of future SMIs for teachers.

## Appendix

Multimedia Appendix 1 More detailed information on the sample and results.

Multimedia Appendix 2 CONSORT-eHEALTH checklist (V 1.6.1).

## Abbreviations

ANCOVA: analysis of covariance
CONSORT: Consolidated Standards of Reporting Trials
CRM: classroom management
CSQ-I: Client Satisfaction Questionnaire adapted for internet-based interventions
ERSQ: Emotion Regulation Skills Questionnaire
IG: intervention group
iSMI: internet-based stress management intervention
ITT: intention-to-treat
NNT: number needed to treat
PSS-10: Perceived Stress Scale
RCT: randomized controlled trial
SMI: stress management intervention
SPSI-R: revised version of the Social Problem-Solving Inventory
WLG: waitlist control group

## Data Availability

The datasets generated or analyzed during this study are available from the corresponding author on reasonable request. The evaluated internet-based stress management intervention is freely available for students of education as part of their health psychology course at Leuphana University of Lüneburg.
