# Supplementary material for: Efficacy of a Web-Based Stress Management Intervention for Beginning Teachers on Reducing Stress and Mechanisms of Change: Randomized Controlled Trial
Source: J Med Internet Res. 2025 Jun 16;27:e58475. doi: 10.2196/58475 (PMC12209733; doi:10.2196/58475)
Supplement: Multimedia Appendix 1 [file jmir_v27i1e58475_app1.docx]

| **Efficacy of a web-based stress management intervention for beginning teachers and mechanisms of change: results of a randomized controlled trial**  Heckendorf & Lehr  **Multimedia Appendix** |
| --- |

**Content**

- Table S1 Internal consistencies of the variables at all assessment points
- Table S2 Effort reward imbalance in the sample at baseline – intention to treat sample
- Table S3 Psychopathological symptoms in the sample at baseline
- Table S4 Further characteristics of the sample
- Table S5 Satisfaction with the training received as measured with the CSQ-I.
- Table S6 Overview of completed CRM sessions and rated usefulness and easiness of each session
- Table S7 Transfer and usage of strategies learned in daily life, assessed at 3-MFU in the intervention group
- Table S8 Results of within-subjects ANOVAs and Cohen’s ds for primary and secondary outcomes between baseline and 6-MFU– intention-to-treat sample
- References

## Table S1

*Internal consistencies of the variables at all assessment points.*

| Outcome | |  | T1 |  | T2 |  | 3-MFU |  | 6-MFU |
| --- | --- | --- | --- | --- | --- | --- | --- | --- | --- |
| Primary outcome | | | | | | | | | |
|  | Perceived Stress |  | 0.71 |  | 0.89 |  | 0.91 |  | 0.89 |
| Secondary outcomes | | | | | | | | | |
| General mental health | | | | | | | | | |
|  | Depression |  | 0.89 |  | 0.9 |  | 0.92 |  | 0.9 |
|  | Anxiety |  | 0.80 |  | 0.89 |  | 0.89 |  | 0.89 |
|  | Insomnia |  | 0.86 |  | 0.88 |  | 0.88 |  | 0.82 |
| Work related outcomes | | | | | | | | | |
|  | Emotional Exhaustion |  | 0.81 |  | 0.88 |  | 0.89 |  | 0.87 |
|  | Work-related rumination |  | 0.88 |  | 0.94 |  | 0.91 |  | 0.88 |
|  | Work related anxiety |  | 0.81 |  | 0.86 |  | 0.89 |  | 0.93 |
|  | Job satisfaction |  | 0.86 |  | 0.87 |  | 0.89 |  | 0.89 |
|  | Effort |  | 0.4 |  | 0.56 |  | 0.6 |  | 0.63 |
|  | Reward |  | 0.69 |  | 0.72 |  | 0.75 |  | 0.78 |
| Coping orientation and self-efficacy | | | | | | | | | |
|  | Emotion-regulation skills |  | 0.82 |  | 0.87 |  | 0.91 |  | 0.88 |
|  | Problem-solving ability |  | 0.84 |  | 0.86 |  | 0.89 |  | 0.85 |
|  | Classroom Management self-efficacy |  | 0.86 |  | 0.91 |  | 0.92 |  | 0.94 |
|  | Work-related self-efficacy |  | 0.78 |  | 0.84 |  | 0.86 |  | 0.84 |

## Table S2

*Effort reward imbalance in the sample at baseline – intention to treat sample*

|  |  | T1 | | | | |  | T2^a^ | | | | |  | 3-MFU^a^ | | | | |  | 6-MFU^a^ | |
| --- | --- | --- | --- | --- | --- | --- | --- | --- | --- | --- | --- | --- | --- | --- | --- | --- | --- | --- | --- | --- | --- |
| _ |  | IG | | _ | WLG | | _ | IG | | _ | WLG | | _ | IG | | _ | WLG | | _ | IG | |
| Outcome | | *N* | % |  | *N* | % |  | *N* | % |  | *N* | % |  | *N* | % |  | *N* | % |  | *N* | % |
| ERI-cut-off > 1^a^ | | | | | | | | | | | | | | | | | | | | | |
|  | ERI-Ratio > 1 | 79 | 79 |  | 69 | 69 |  | 61 | 61 |  | 76 | 76 |  | 57 | 57 |  | 77 | 77 |  | 43 | 43 |
|  | ERI-Ratio ≤ 1 | 21 | 21 |  | 31 | 31 |  | 39 | 39 |  | 24 | 24 |  | 43 | 43 |  | 23 | 23 |  | 57 | 57 |
| ERI-cut-off > 0.715 ^b^ | |  |  |  |  |  |  |  |  |  |  |  |  |  |  |  |  |  |  |  |  |
|  | ERI-Ratio > 0.715 | 97 | 97 |  | 99 | 99 |  | 95 | 95 |  | 97 | 97 |  | 91 | 91 |  | 95 | 95 |  | 79 | 79 |
|  | ERI-Ratio ≤ 0.715 | 3 | 3 |  | 1 | 1 |  | 5 | 5 |  | 3 | 3 |  | 9 | 9 |  | 5 | 5 |  | 21 | 21 |

*Note.* IG = intervention group; WLG = wait list control group; T1 = baseline; T2 = post-intervention; 3-MFU = 3-month follow-up; 6-MFU = 6-month follow-up. The 6-MFU was only assessed in the IG; ERI = Effort-Reward-Imbalance.

^a^ cut-off score according to the theoretical conceptualization by (Siegrist et al., 2004), ^b^ empirically derived cut-off according to Receiver-operating characteristic analyses (Lehr et al., 2010).

## Table S3

*Psychopathological symptoms in the sample at baseline*

|  | |  | Total  (*N* = 200) | |  | IG  (*n* = 100) | |  | WLG  (*n* = 100) | |
| --- | --- | --- | --- | --- | --- | --- | --- | --- | --- | --- |
|  | |  | *N* | % |  | *n* | % |  | *n* | % |
| Depressive symptoms | | |  |  |  |  |  |  |  |  |
|  | <18 | | 82 | 41 |  | 34 | 34 |  | 48 | 48 |
|  | ≥ 18 | | 118 | 59 |  | 66 | 66 |  | 52 | 52 |
| Anxiety symptoms | | | | | | | | | | |
|  | < 10 | | 82 | 41 |  | 39 | 39 |  | 43 | 43 |
|  | ≥ 10 | | 118 | 59 |  | 61 | 61 |  | 57 | 57 |
| Insomnia symptoms | | |  |  |  |  |  |  |  |  |
|  | < 15 | | 123 | 61.5 |  | 55 | 55 |  | 68 | 68 |
|  | ≥ 15 | | 77 | 38.5 |  | 45 | 45 |  | 32 | 32 |

*Note.* For depressive symptoms a score ≥ 18 indicates clinically-significant levels of depression. For anxiety symptoms a score ≥ 10 indicates moderate to severe levels of anxiety. For insomnia symptoms a total score ≥ 15 indicates moderate to severe levels of insomnia.

## Table S4

*Further characteristics of the sample*

|  | |  | N |  | % |
| --- | --- | --- | --- | --- | --- |
| Grades taught | |  |  |  |  |
|  | 1-4 |  | 57 |  | 28.5 |
|  | 5-6 |  | 69 |  | 34.5 |
|  | 7-10 |  | 118 |  | 59 |
|  | 11-13 |  | 84 |  | 42 |
| Classes taught | |  |  |  |  |
|  | German |  | 76 |  | 38 |
|  | Math |  | 53 |  | 26.5 |
|  | English |  | 37 |  | 18.5 |
|  | General studies |  | 31 |  | 15.5 |
|  | Art |  | 25 |  | 12.5 |
|  | Biology |  | 22 |  | 11 |
|  | History |  | 21 |  | 10.5 |
|  | Religion |  | 20 |  | 10 |
|  | Other Subjects |  | 128 |  | 64 |
| Federal state | |  |  |  |  |
|  | North Rhine-Westphalia |  | 49 |  | 24.5 |
|  | Bavaria |  | 30 |  | 15.0 |
|  | Lower Saxony |  | 27 |  | 13.5 |
|  | Baden-Wuerttemberg |  | 23 |  | 11.5 |
|  | Hesse |  | 18 |  | 9.0 |
|  | Berlin |  | 13 |  | 6.5 |
|  | Saxony |  | 8 |  | 4.0 |
|  | Other |  | 32 |  | 16 |

## Table S5

*Satisfaction with the training received as measured with the CSQ-I.*

| Item | Does not apply | Does not really apply | Partly applies | Fully applies |
| --- | --- | --- | --- | --- |
| The training I attended was of high quality | 0% | 2.8% | 31.9% | 65.3% |
| I received the kind of training I wanted | 1.4% | 8.3% | 55.6% | 34.7% |
| The training has met my needs | 1.4% | 2.8% | 65.3% | 30.6% |
| I would recommend this training to a friend, if he or she were in need of similar help | 2.8% | 6.9% | 26.4% | 63.9% |
| I am satisfied with the amount of help I received through the training | 1.4% | 6.9% | 47.2% | 44.4% |
| The training helped me deal with my problems effectively | 1.4% | 9.7% | 44.4% | 44.4% |
| In an overall, general sense, I am satisfied with the training | 1.4% | 1.4% | 37.5% | 59.7% |
| I would come back to such a training if I were to seek help again | 2.8% | 8.3% | 29.2% | 59.7% |

## Table S6

*Overview of completed CRM sessions and rated usefulness and easiness of each session*

| CRM session | |  |  | CRM 1 | |  | CRM 2 | |  | CRM 3 | |  | CRM 4 | |  | CRM 5 | |  | CRM 6 | |  | CRM 7 | |
| --- | --- | --- | --- | --- | --- | --- | --- | --- | --- | --- | --- | --- | --- | --- | --- | --- | --- | --- | --- | --- | --- | --- | --- |
|  | |  |  | *N* | % |  | *N* | % |  | *N* | % |  | *N* | % |  | *N* | % |  | *N* | % |  | *N* | % |
| Completed session | |  |  | 56 | 56.0 |  | 29 | 29.0 |  | 43 | 43.0 |  | 17 | 17.0 |  | 14 | 14.0 |  | 18 | 18.0 |  | 6 | 6.0 |
| Gave feedback within session | | |  | 43 | 76.8 |  | 27 | 93.1 |  | 18 | 41.9 |  | 12 | 70.6 |  | 11 | 78.6 |  | 11 | 61.1 |  | 6 | 100.0 |
| General usefulness^1^ | |  |  |  |  |  |  |  |  |  |  |  |  |  |  |  |  |  |  |  |  |  |  |
|  | not at all |  |  | 0 | 0.0 |  | 0 | 0.0 |  | 0 | 0.0 |  | 0 | 0.0 |  | 0 | 0.0 |  | 0 | 0.0 |  | 0 | 0.0 |
|  | rather not |  |  | 5 | 11.6 |  | 8 | 29.6 |  | 2 | 11.1 |  | 0 | 0.0 |  | 0 | 0.0 |  | 1 | 9.1 |  | 1 | 16.7 |
|  | moderately |  |  | 5 | 11.6 |  | 10 | 37.0 |  | 2 | 11.1 |  | 2 | 16.7 |  | 2 | 18.2 |  | 0 | 0.0 |  | 2 | 33.3 |
|  | rather yes |  |  | 19 | 44.2 |  | 7 | 25.9 |  | 10 | 55.6 |  | 5 | 41.7 |  | 5 | 45.5 |  | 6 | 54.5 |  |  | 0.0 |
|  | very |  |  | 14 | 32.6 |  | 2 | 7.4 |  | 4 | 22.2 |  | 5 | 41.7 |  | 4 | 36.4 |  | 4 | 36.4 |  | 3 | 50.0 |
| Easy to complete^2^ | |  |  |  | 0.0 |  |  | 0.0 |  |  | 0.0 |  |  | 0.0 |  |  | 0.0 |  |  | 0.0 |  |  | 0.0 |
|  | not at all |  |  | 0 | 0.0 |  | 0 | 0.0 |  | 0 | 0.0 |  | 0 | 0.0 |  | 0 | 0.0 |  | 0 | 0.0 |  | 0 | 0.0 |
|  | rather not |  |  | 1 | 2.3 |  | 1 | 3.7 |  | 1 | 5.6 |  | 1 | 8.3 |  | 0 | 0.0 |  | 0 | 0.0 |  | 0 | 0.0 |
|  | moderately |  |  | 2 | 4.7 |  | 4 | 14.8 |  | 2 | 11.1 |  |  | 0.0 |  | 0 | 0.0 |  | 0 | 0.0 |  | 0 | 0.0 |
|  | rather yes |  |  | 16 | 37.2 |  | 8 | 29.6 |  | 6 | 33.3 |  | 5 | 41.7 |  | 4 | 36.4 |  | 2 | 18.2 |  | 1 | 16.7 |
|  | very |  |  | 24 | 55.8 |  | 14 | 51.9 |  | 9 | 50.0 |  | 6 | 50.0 |  | 7 | 63.6 |  | 9 | 81.8 |  | 5 | 83.3 |
| *Note.* CRM = classroom management. For the particular content of each session see Table 1 in the OSM. ^1^ Was this session of the optional CRM-module useful for you? ^2^ Was this session of the optional CRM-module easy for you to go through? | | | | | | | | | | | | | | | | | | | | | | | |

## Table S7

*Transfer and usage of strategies learned in daily life, assessed at 3-MFU in the intervention group*

|  | Not at all |  | Rarely |  | Sometimes |  | More often |  | Almost daily |  | Do not remember |
| --- | --- | --- | --- | --- | --- | --- | --- | --- | --- | --- | --- |
| Positive/recreational activities | 0 |  | 3 (5.2%) |  | 8 (13.8%) |  | 23 (39.7%) |  | 23 (39.7%) |  | 1 (1.7%) |
| Problem solving strategies | 7 (12.1%) |  | 11 (19.0%) |  | 19 (32.8%) |  | 12 (20.7%) |  | 5 (8.6%) |  | 4 (6.9%) |
| Relaxation exercises | 5 (8.6%) |  | 8 (13.8%) |  | 13 (22.4%) |  | 18 (31.0%) |  | 8 (13.8%) |  | 6 (10.3%) |
| Strategies for acceptance of emotions | 8 (13.8%) |  | 2 (3.4%) |  | 20 (34.5%) |  | 13 (22.4%) |  | 4 (6.9%) |  | 11 (19.0%) |
| Strategies for self-support | 2 (3.4%) |  | 8 (13.8%) |  | 9 (15.5%) |  | 17 (29.3%) |  | 10 (17.2%) |  | 12 (20.7%) |
| Teacher-specific individualized modules | 6 (10.3%) |  | 8 (13.8%) |  | 9 (15.5%) |  | 17 (29.3%) |  | 5 (8.6%) |  | 13 (22.4%) |
| Non-occupation specific individualized modules^a^ | 3 (5.2%) |  | 7 (12.1%) |  | 15 (25.9%) |  | 16 (27.6%) |  | 10 (17.2%) |  | 7 (12.1%) |

*Note.* Answering the following item: “To what extent have you been able to use the following strategies in your daily life since the end of the training?”

^a^ Strategies from the Info-Corner included: Time management, consciously planning breaks, switching off from work, strategies for reducing brooding and worry, sleeping better, social support, nutrition, and exercise

## Table S8.

*Results of within-subjects ANOVAs and Cohen’s ds for primary and secondary outcomes between baseline and 6-MFU– intention-to-treat sample*

|  |  |  | | | | | | |  | Differences within IG | | |
| --- | --- | --- | --- | --- | --- | --- | --- | --- | --- | --- | --- | --- |
|  |  |  | | |  |  | | |  | 6-MFU | | |
| Outcome | |  |  |  |  |  |  |  |  | *F_df_* | *P* | Cohen’s *d*  [95% CI] ^a^ |
| Primary outcome | | | | | | | | | | | | |
|  | Perceived Stress |  |  |  |  |  |  |  |  | 76.9_1,43_ | <.001 | 1.57 [1.16,1.95] |
| Secondary outcomes | | | | | | | | | | | | |
| Mental health | | | | | | | | | | | | |
|  | Depression |  |  |  |  |  |  |  |  | 32.2_1,62_ | <.001 | 0.87 [0.58,1.15] |
|  | Anxiety |  |  |  |  |  |  |  |  | 39.5_1,53_ | <.001 | 1.00 [0.71,1.28] |
|  | Insomnia |  |  |  |  |  |  |  |  | 36.4_1,33_ | <.001 | 1.02 [0.77,1.24] |
| Work-related health | | | | | | | | | | | | |
|  | Emotional Exhaustion |  |  |  |  |  |  |  |  | 26.1_1,26_ | <.001 | 0.99 [0.75,1.21] |
|  | Work-related rumination |  |  |  |  |  |  |  |  | 44.6_1,29_ | <.001 | 1.23 [0.94,1.50] |
|  | Work-related anxiety |  |  |  |  |  |  |  |  | 13.5_1,29_ | <.001 | 0.70 [0.47,0.92] |
|  | Job Satisfaction |  |  |  |  |  |  |  |  | 8.1_1,35_ | .005 | 0.36 [0.21,0.51] |
|  | Effort |  |  |  |  |  |  |  |  | 25.4_1,34_ | <.001 | 1.01 [0.72,1.29] |
|  | Reward |  |  |  |  |  |  |  |  | 0.33_1,24_ | .57 | 0.13 [-0.06,0.31] |
|  | Absenteeism days^b,c^ |  |  |  |  |  |  |  |  | 0.66_1,51_ | .42 | 0.16 [-0.23,0.55] |
|  | Presenteeism days^b,c^ |  |  |  |  |  |  |  |  | 5.07_1,51_^*^ | .003 | 0.40 [0.05,0.74] |
| Coping and self-efficacy | |  |  |  |  |  |  |  |  |  |  |  |
|  | Emotion-regulation skills |  |  |  |  |  |  |  |  | 57.2_1,52_ | <.001 | 1.08 [0.80,1.35] |
|  | Problem-solving ability |  |  |  |  |  |  |  |  | 54.4_1,43_ | <.001 | 1.20 [0.86,1.52] |
|  | CRM self-efficacy |  |  |  |  |  |  |  |  | 18.0_1,34_ | <.001 | 0.58 [0.42,0.74] |
|  | Work-related self-efficacy |  |  |  |  |  |  |  |  | 16.0_1,33_ | <.001 | 0.62 [0.41,0.81] |

*Note.* IG = intervention group; WLG = waiting list control group; 6-MFU = 6-month follow-up; CI = Confidence Interval; CRM=Classroom Management.

^a^ Cohen’s *d*s for within-subject effects were calculated by controlling for dependence within samples. ^b^Missing values were not imputed for absenteeism or presenteeism days. ^c^Absenteeism and presenteeism days during the past three months for T1, 3-MFU and 6-MFU.

# References:

Lehr, D., Koch, S., & Hillert, A. (2010). Where is (im)balance? Necessity and construction of evaluated cut-off points for effort-reward imbalance and overcommitment. *Journal of Occupational and Organizational Psychology*, *83*(1), 251–261. https://doi.org/10.1348/096317909X406772

Siegrist, J., Starke, D., Chandola, T., Godin, I., Marmot, M., Niedhammer, I., & Peter, R. (2004). The measurement of effort–reward imbalance at work: European comparisons. *Social Science & Medicine*, *58*(8), 1483–1499. https://doi.org/10.1016/S0277-9536(03)00351-4
